# Public health performance of sanitation technologies in Tamil Nadu, India: Initial perspectives based on *E. coli* release

**DOI:** 10.1016/j.ijheh.2022.113987

**Published:** 2022-06

**Authors:** Musa Manga, Pete Kolsky, Jan Willem Rosenboom, Sudha Ramalingam, Lavanya Sriramajayam, Jamie Bartram, Jill Stewart

**Affiliations:** a The Water Institute at UNC, Department of Environmental Sciences and Engineering, The Gillings School of Global Public Health, University of North Carolina at Chapel Hill, 4114 McGavran Hall, Campus Box # 7431, NC, 27599, Chapel Hill, NC, USA; b Bill & Melinda Gates Foundation, Seattle, WA, USA; c PSG Institute of Medical Sciences and Research, Off, Avinashi Rd, Masakalipalayam, Peelamedu, Coimbatore, Tamil Nadu, 641004, India; d School of Civil Engineering, University of Leeds, Leeds, LS2 9JT, UK; e Department of Construction Economics and Management, College of Engineering, Design, Art and Technology (CEDAT), Makerere University, P.O. Box 7062, Kampala, Uganda

**Keywords:** Pathogen flow, Containment system typology, Septic tanks, Pathogen release, Septic system design, Faecal sludge management

## Abstract

Sanitation is intended to reduce the spread and burden of diseases transmitted from excreta. Pathogen reduction from excreta before sludge or effluent discharge to the environment would seem a logical and useful performance indicator for sanitation systems. However, the relative magnitudes of pathogen release from common sanitation technologies are not well understood. We, therefore, investigated the feasibility of performance measurement of different sanitation technologies in Tamil Nadu, India in reducing the release of the pathogen indicator *Escherichia coli* (*E. coli)*. After conducting users’ surveys and technical assessments of the locally prevalent sanitation systems, we classified them into 7 distinct categories (based on both observed physical characteristic and usage) within a widely-accepted physical typology. Faecal sludge and wastewater samples were collected and analysed for *E. coli* and total solids from 136 household systems, 24 community systems, and 23 sanitary sewer oveflows. We estimated the average volumetric release rates of wastewater and faecal sludge from the different sanitation technologies. Average daily per capita *E. coli* release was computed, and used as one indicator of the public health performance of technologies. We found that on-site installations described by owners as “septic systems” included diverse forms of tanks and pits of uncertain performance. We observed a statistically significant difference in the average daily per capita *E. coli* release from different sanitation technologies (*p* = 0.00001). Pathogen release from the studied on-site sanitation technologies varied by as much as 5 orders of magnitude from “lined pits” (5.4 Log10 *E. coli* per person per day) to “overflowing sanitary sewers” and “direct discharge pipes” (10.3–10.5 Log10 *E. coli* per person per day). Other technologies lay between these extremes, and their performances in *E. coli* removal also varied significantly, in both statistical and practical terms. Our results suggest that although faecal sludge management along the sanitation service chain is important, sanitation planners of the observed systems (and probably elsewhere) should direct higher priority to proper management of the liquid effluents from these systems to minimize public health hazards. We conclude that (i) the work demonstrates a new and promising approach for estimating the public health performance of differing sanitation technologies, (ii) if *E.coli* is accepted as an indicator of the public health hazard of releases from sanitation systems, our results strongly suggest that safe containment of excreta for an extended period substantially reduces pathogen numbers and the risk of pathogen release into the environment; and (iii) there are some simple but little-used technical improvements to design and construction of on-site sanitation systems which could significantly reduce the release of pathogens to the environment.

## 1 Introduction

Sanitation-related diseases caused by exposure to faecal pathogens (including various bacteria, viruses, helminths, and protozoa) cause a substantial global burden of disease including 1.7 billion episodes of diarrhoea every year in children under 5 years (Walker et al., 2013). Access to adequate sanitation alone would eliminate about 0.5 million deaths and 26 million DALYs of diarrhoea every year, especially in the Low and Middle Income Countries (Prüss-Ustün et al., 2019).

Communities everywhere include households with diverse individual and collective ways of managing excreta. It has been estimated that over 3.1 billion people globally rely on household on-site sanitation facilities (pit latrines, cesspits and diverse “septic systems”) (UNICEF and WHO, 2019), and this population is anticipated to increase to 5 billion by 2030 (Strande and Brdjanovic, 2014). The on-site systems function by containing excreta, either in a pit latrine (which receives excreta with minimal water until it is filled, when its contents are emptied as faecal sludge) or in some form of “septic system” (which allows for the management of large amounts of wastewater without necessarily spilling directly into the local environment). In this paper the term “septic system” in quotation marks refers to any of a wide variety of poorly designed and operated on-site sanitation systems which receives wastewater, stores septage, and discharges liquid effluent to the environment. In contrast, the term septic system *without* quotation marks refers to a much narrower, and rarer, subset of well-designed and operated systems which meet common widely accepted engineering design criteria for septic systems to improve performance. The term pit refers to an on-site sanitation system which receives excreta or wastewater into a hole, and stores faecal sludge as the liquid fraction ex-filtrates into the surrounding soils.

In India, about 45% of the urban households (approximately 600 million people (Plecher, 2020)) are served by on-site sanitation systems –mainly “septic systems” (Census of India, 2011; Rohilla et al., 2016b). In Urban Tamil Nadu, India, around 38% of households use “septic systems” for their sanitation needs, 27% are connected to sanitary sewers, and 35% use others (such as pit latrines (6.0%), shared facilities (9.9%), direct discharge pipes (1.2%), open defecation (16.5%), etc.) (Census of India, 2011; IIPS and ICF, 2017). This range of sanitation technologies and service chains poses practical and important questions for sanitation managers. Which is a greater public health priority in a given city: reduction of covert faecal sludge dumping from “septic systems” and pit latrines, or better wastewater treatment? Reduction of the immediate direct discharge of black-water to the environment or open defecation by a small fraction of the population, or better treatment or control of “septic system” effluent discharged by many? Such decisions should reflect the relative benefits, costs, reliability, and operation and maintenance requirements of different technologies, which all vary with local conditions.

In principle, a septic system consists of both (i) a well-designed watertight chamber (i.e. fully-lined tank) that receives domestic wastewater for basic treatment through sedimentation and anaerobic processes to reduce organics and total solids; and (ii) the effluent receiver (such as a drain field, etc.) for further treatment and disposal of the tank effluent (Missouri DHSS., 2018; Georgia Department of Public Health, 2019; Feachem et al., 1981; Wang et al., 2021; Koottatep et al., 2014). However, the design, construction, operation, and maintenance of septic systems are not well understood by users, policy makers, and utility authorities especially in the global south. This confusion has resulted in a chaotic mixture of poorly designed and constructed tanks/on-site sanitation systems for management of excreta, with widely varying effluent quality and disposal practice, with little or no concern for public health (Strande et al., 2018).

How can we begin to estimate the effectiveness (or public health threats) from diverse sanitation technologies in a community without understanding the pathogen inactivation and releases of these systems? Without measurement and analysis, the effects of different design, construction, and operational features of these systems on pathogen inactivation and release will remain poorly understood. While the types and typical concentrations of pathogens present in excreta have been documented (e.g. Feachem et al. (1981); Harwood et al. (2017); Penn et al. (2018)), the pathogen releases in liquid effluent or emptied faecal sludge from on-site sanitation systems, remain scarcely characterised (Williams and Overbo, 2015; Wang et al., 2021; Foster et al., 2021; Amin et al., 2020; Manga et al., 2019, Manga et al., 2021, Manga et al., 2016).

The problem is complex because faecal sludge and effluent from on-site sanitation systems vary substantively, depending upon factors including type of containment, detention time, desludging practice, quality of construction, household usage, and operation of the system. Previous studies on the performance of on-site sanitation technologies (especially “septic systems”) focus on removal of physical-chemical pollutant indicators (e.g. pH, conductivity, total suspended solids, biochemical oxygen demand, algal nutrients, etc.) from liquid effluent or faecal sludge (Abbassi et al., 2018; Bounds, 1997; Burubai et al., 2007; Levett et al., 2010; Nasr and Mikhaeil, 2013; Philippi et al., 1999; Rich et al., 2004; Strande et al., 2018; Prasad et al., 2021; Englund et al., 2020). However, studies of pathogen reduction in septic systems are few. Some studies demonstrate that the fully-lined tanks of the septic systems act as primary treatment units for solids removal from wastewater, reducing *E. coli* concentrations by 1–2 Log_10_ – mainly through sedimentation (Abbassi et al., 2018; Pfluger et al., 2009; Stenström et al., 2011; Brandes, 1978; Wang et al., 2021); however these studies do not account for the release of *E. coli* in emptied faecal sludge and/or liquid discharge.

We set out to investigate (i) the characteristics and key design features of local on-site sanitation systems, (ii) relative *E. coli* concentrations and average daily volumetric discharges of excreta from different sanitation technologies, (iii) relative *E. coli* releases from different sanitation technologies in liquid discharges and/or faecal sludge removal through periodic desludging, (iv) the effectiveness of local sanitation technologies in reducing *E. coli* release to the environment and/or the next stage of the sanitation service chain, and (v) the effect of key design, construction, and operational features of these systems on their performance in terms of *E. coli* release. To meet these objectives, we collected, synthesised and analysed field data from community transect walks, household interviews, key informant interviews, and observational surveys, technical assessments, and environmental sampling of locally prevalent sanitation technologies from two study sites within Tamil Nadu, India.

## 2 Methods

### 2.1 Study areas

Our study was conducted in two urban communities in the south Indian state of Tamil Nadu: (i) the Town Panchayat of Narasimhanaicken-Palayam (NNP) in Coimbatore district at an altitude of 473 m above mean sea level (geographical coordinates 11°7′31.44″ N latitude, 76°55′33.24″ E longitude), and (ii) Tiruchirappalli (Trichy) City Corporation (TCC) at altitude of 88 m above mean sea level (geographical coordinates 10°48′18″ N latitude, 78°41′8.16″ E longitude). Both communities have been supported by the TNUSSP - Tamil Nadu Urban Sanitation Support Programme (TNUSSP is a Technical Support Unit set up to support the Govt. of Tamil Nadu in scaling up urban sanitation across the state. It is a consortium, in which the Indian Institute for Human Settlements is the lead partner). These sites reflect a spectrum of excreta return pathways from common sanitation technologies in Tamil Nadu (Tiruchirappalli City Corporation, 2018; TNUSSP, 2018a; TNUSSP, 2017; TNUSSP, 2016). These communities use similar sanitation technologies, including various on-site sanitation systems discharging liquid effluent, although NNP lacks the municipal sewerage and wastewater treatment found in Trichy. The communities we selected differ in scale—a major city (Trichy) versus a smaller urban administrative unit (NNP) on the outskirts of the major city of Coimbatore. The sanitation service chains for these sites also differ, with, for example, municipal faecal sludge decanting stations in Trichy, in contrast to the widespread direct agricultural reuse of untreated faecal sludge just outside NNP.

Sanitation challenges common to both study communities include: households without access to individual toilets, and a high proportion of households dependant on poorly designed and constructed on-site sanitation systems (pits and tanks) with no proper faecal sludge management services (TNUSSP, 2018a; TNUSSP, 2017; TNUSSP, 2016). The small sewerage system of Trichy is inadequately operated and managed and has insufficient wastewater and faecal sludge treatment capacity.

### 2.2 Community transect walks and key informant interviews

Field work for this cross-sectional study was conducted between March 2018 and October 2019. Initially, we conducted key informant interviews with the stakeholders along the sanitation service chain (such as pit emptiers, managers of community toilets, operators of sanitary sewers and FS treatment facilities, etc.). In addition to these interviews, we conducted 20 transect walks in Trichy and 10 transect walks in NNP to identify the different sanitation technologies used in the study area.^1^ All community transect walks were conducted using well-tested and documented methods (World Bank Group (2016). The route for each transect walk was identified, discussed, and agreed on by all the participants at least a day in advance. Such routes crossed the study communities following a winding path to include a variety of areas that represent the study area. The route taken for each transect walk was planned and recorded using GPS data.

### 2.3 Sanitation Technology Typology in the study communities

**Fig. 1:**
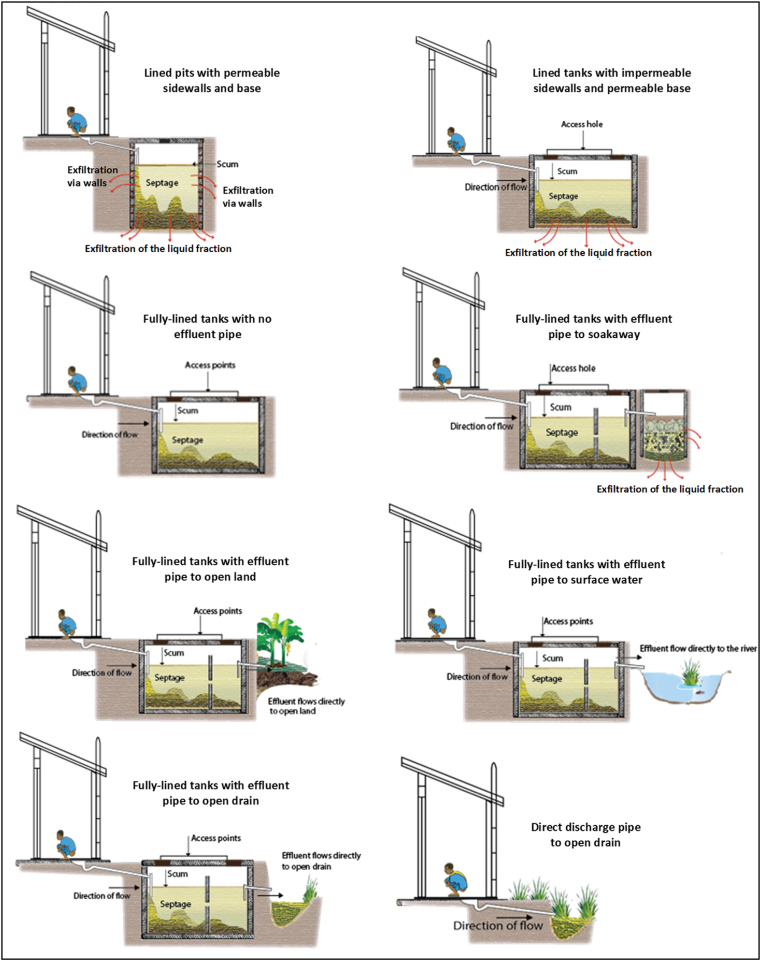
Common sanitation technologies in the study area.

Fig. 1 illustrates the Sanitation Technology Typology by the structural characteristics of the different systems identified in the study communities, based on the work of the SFD Promotion Initiative (2017). Table 1 describes the different routes through which these sanitation technologies release *E. coli* (and other pathogens) to the environment and/or next stage of the sanitation service chain. The typology used thus classifies sanitation technologies by physical measurements and observation to identify relevant pathogen release mechanisms for each system.

**Table 1:** Typology of sanitation used in this study. (adapted from SFD Promotion Initiative (2017)).

Technology Type	Structural characteristics of tank or system	Release of *E. coli* (and other pathogens) from sanitation systems		
		Discharge		Septage/sludge removal by tank emptiers
		Effluent to open ground, surface water, or open-drain	Overflows to environment	
Lined Pit	Permeable sidewall and base	No	No	Yes
Lined Tank	Impermeable sidewalls and permeable base	No	No	Yes
Fully-Lined Tank *without* effluent pipe	Impermeable sidewalls and base	No	No	Yes
Fully-Lined Tank *with* effluent pipe		Yes	No	Yes
Community Toilet Fully-lined Tank (Fully-Lined Tank with effluent pipe, shared by multiple households)		Yes	No	Yes
Direct Discharge Pipes (also known as “straight pipes”, “black-water pipes”)	Short pipe discharging directly to the environment	No	Yes, constantly	No
Sanitary Sewers	Pipes carrying wastewater to the municipal network	No	Yes, at locations where sewers are broken or often blocked	No

We used a “mass balance” approach to explore the release of *E. coli* from household sanitation systems: In this approach, *E. coli* enter the system with excreta, and are removed either by (1) liquid discharge (in releases of effluent, or overflows) or exfiltration, (2) die-off during treatment or storage in the system, or (3) faecal sludge or septage removal through periodic desludging. Effluent refers to the liquid discharge from on-site sanitation systems (for example, a tank with discharge pipes) to open ground, surface water, or open drain. By contrast, overflow refers to (i) sewage overflows at parts of the sanitary sewer associated with frequent blockages and overflows, or (ii) direct discharge without treatment (i.e. direct discharge pipes) to the open drains or environment. Exfiltration refers to seepage through the floor or side walls of the tank, pit and/or discharge to a soakaway. These technologies are illustrated in Fig. 1.

As indicated in Table 1 and shown in Fig. 1, a **lined pit** is constructed with an open bottom and permeable linings (e.g. honeycombed lined walls or perforated pre-cast concrete rings) through which exfiltration can occur into the surrounding soils. A **lined tank** refers to a tank constructed with impermeable sidewalls and permeable bottom/base, while a **fully-lined tank** is constructed with both impermeable sidewalls and base (See Fig. 1). In this paper the term **decanting station** refers to a designated facility through which faecal sludge is discharged into the sanitary sewer that conveys it with sewage to the wastewater treatment plant (TNUSSP, 2018b).

### 2.4 Selection of sanitation systems for study

An inventory of all the sanitation systems identified in the study communities based on community visits and key informant interviews was prepared, and used as a sampling frame. The final selection of the sanitation systems for detailed technical review and effluent, septage, and faecal sludge sampling was purposive and reflected a balance of criteria: (i) logistical or resource constraints; (ii) accessibility of the containment systems and sampling points for on-site visual assessment and sample collection; and (iii) the willingness of the system owners and users to have their systems included in the study. The results are shown in Table 2.

**Table 2:** Distribution of observed and studied sanitation systems by technology in Trichy, and NNP.

Study Areas	Row Total of Sites	Household Fully-lined Tanks	Lined Pits	Lined Tanks	Community Fully-lined Tanks	Direct Discharge Pipes	Sanitary Sewer Discharges
Trichy Inventory	465	260	26	15	37	80	47
NNP Inventory	88	12	43	20	13	N/A	N/A
**Selection for Initial Site Surveys**							
In Trichy	183	105	2	10	18	25	23
In NNP	43	1	17	15	10	N/A	N/A
**Total in both Trichy and NNP**	**226**	**106**	**19**	**25**	**28**	**25**	**23**
**Final Selection for Detailed Surveys and Environmental Sampling**							
In Trichy	148	**74**	2	10	14	25	23
In NNP	35	1	13	11	10	N/A	N/A
**Total in both Trichy and NNP**	**183**	**75**	**15**	**21**	**24**	**25**	**23**

N/A – Not applicable as that sanitation system was not found in the study area.

### 2.5 Field worker training and piloting of data collection tools

Experienced local research assistants and enumerators with knowledge of WaSH, expertise of using mWater (New York, USA) mobile platform and fluent in English and the local language (Tamil), were recruited and trained in data collection for this study in a five-day facilitated workshop.

The surveys were independently checked, pretested during surveyor training, and piloted in non-study villages. Rigorous testing and retesting of the surveys were carried out during programming of the electronic survey in mWater.

### 2.6 Initial site surveys

Prior to detailed surveys and environmental sampling, the initial site data were collected through user surveys and technical surveys at each of the selected sanitation systems. The user and observation survey questions were communicated by the enumerators in either English or the local language (Tamil), as preferred by the respondent. Data collected through these surveys were captured electronically using the mWater data collection platform, using Android-enabled smartphones or tablets.

#### 2.6.1 User (household) surveys

A total of 203 user surveys were conducted with household owners or users of the 178 containment systems and 25 direct discharge pipes in the study. Users were asked about (i) the number of users of the containment systems, (ii) the emptying frequency over the past 5 years, (iii) the date when the containment system was last emptied and (iv) primary means of greywater disposal (where greywater is discharged to open drain, open ground, containment system, soakaways or others). A complete list of user survey questions is provided in Appendix C (Table S10) of the Supplementary Material. These data were used to (i) guide the technical assessment of the containment system; (ii) estimate storage periods between desludging; and (iii) permit analysis of the field measurements and samples collected from the studied systems.

#### 2.6.2 Technical surveys

Data were collected by on-site visual inspections of the containment systems, taking measurements as appropriate. Each of the 178 containment systems was surveyed to determine (i) the shape and key design features of the system (e.g. plastic sanitary inlet or outlet tee-pipes, effluent pipes, the number of chambers, and lastly, the inlet and outlet pipe configurations), (ii) the structural integrity and permeability of both sidewalls and bottom of the containment systems and (iii) black-water or effluent receivers. During the last round of sampling, the containment systems were fully emptied and re-inspected for features that may have been missed or not visible in previous inspections. The collected observational data were used to guide the subsequent physical design, construction, structural assessment and classification of containment systems encountered in the field. The complete observation survey checklist is provided in Appendix C (Table S11) of the Supplementary Material.

The length, width, or diameter of the 178 containment systems in the study were measured using a Bosch Blaze GLM50C Bluetooth Enabled 165-Foot Laser distance measure with color backlit display (Malaysia) and/or an Ironton 44045 steel tape measure 1-inch by 25-Foot (USA). The laser measure tool was calibrated for accuracy using the standardized steel tape measure. The depth of the tank, freeboard, and faecal sludge level were measured using a 12 mm MS dipping stick fabricated locally, and an Ironton 44045 steel tape measure. From these, the effective depth, effective volume of the tank, and actual volume of faecal sludge/septage in the tank were computed. All the key design features observed in the containment system were inspected and their measurements taken.

#### 2.6.3 Quality control of field surveys

Enumerators were audited by the Water Institute staff by resurveying a selection of households and re-inspecting/re-assessing the on-site sanitation systems. Data quality comparison and quality assurance checks and procedures were carried out at multiple stages of data collection.

### 2.7 Detailed surveys and environmental sampling

Detailed surveys and environmental sampling were conducted in two phases for a period of 6 months, the first phase in April–June 2018 and the second phase in August–October 2019 with the intent of capturing seasonal differences (Weather Spark, 2022). Despite the deliberate timing of our field activities for both the usual “rainy” and “dry” seasons, there was no rainfall during our sample collection period, including the six weeks that overlapped with the expected rainy season for 2019. All the environmental sampling activities were conducted in the dry season with average temperatures of about 24–31 °C and humidity of 54%–78% (Weather Spark, 2022). Data collected through physical measurements and environmental sampling of the different sanitation systems were used in computation of *E. coli* releases from the different sanitation systems.

#### 2.7.1 Containment systems

Data were collected from the selected 135 containment systems (75 household fully-lined tanks, 15 lined pits, 21 lined tanks, and 24 community toilet fully-lined tanks) (See Table 2 and Fig. 1). Data collection from each sampling site included a user interview, observational survey, physical measurement, and technical assessment of the containment system.

Effluent and faecal sludge/septage samples were collected from each studied containment system at least 2 times during the study. On each such visit, two types of samples were collected: (i) a single composite sample from the containment system liquid discharge (for systems with effluent pipes to the environment), and (ii) a composite sample of faecal sludge/septage from the containment system (a mixture of samples from the bottom, middle and top of the tank). All effluent and faecal sludge/septage samples were collected from the different containments systems according to sampling procedures in Koottatep et al. (2021), Bassan et al. (2016) and Koottatep et al. (2014).

For the liquid discharge (i.e. effluent) composite sample, 10 grab samples of each 2-L (at an interval of 5 min) were collected from the effluent pipe of the containment system (Koottatep et al., 2014; Nam et al., 2006; Amin et al., 2020). These were then mixed in a sterile container to form a composite sample from which a 1-L aliquot was collected, labelled, and taken to the lab for analysis.

For faecal sludge/septage samples; during the first and/or second round of sampling, composite samples of containment system faecal sludge/septage were collected from each of three depths below the faecal sludge/septage surface: top (between 0.0 and 0.15m); middle (about 0.5–1.0 m); and bottom (about 1.0–1.5 m). At each depth, 10–15 samples of 1-L were collected randomly using an adjustable handle metallic deep-sludge sampler, making a total of between 30 and 45 samples from each containment system. These were then transferred into a sterile container and the samples from each depth were thoroughly mixed to form a composite sample from which a 1-L aliquot was collected and transported to the laboratory for analysis. The metallic deep-sludge sampler was disinfected and sterilized with either bleach and/or 96% alcohol and flamed to sterilize between uses (before and after use). The deep-sludge sampler used in this study was designed and fabricated locally according to Nabateesa et al. (2017) device specifications, but with some modifications to suit the sanitation technologies within the study area. The modifications were aimed at limiting chances of cross-contamination of the collected sample when drawing the equipment from a deeper depth.

During the final round of sampling, faecal sludge samples were collected during a complete emptying of the tank. About 10 grab samples of each 2-L were collected at each of three stages of the emptying process using grab sampling beaker device: (the start, the middle, and the end). The collected grab samples at each stage were mixed to form a composite sample of the faecal sludge being emptied, from which a 1-L aliquot was collected and taken to the laboratory for analysis.

All collected samples were stored in a portable ice chest/cooler box with ice packs and transported to the PSG Institute of Medical Sciences and Research laboratory, where they were stored at below 4 °C until analysis. The average daily discharge as liquid effluent release (q_D_) from the containment systems (in litres/day) was estimated based on one of two ways: (1) the hydrostatic/volume balance and/or (2) a collected volume of liquid discharge (i.e. effluent) in a given time period. The minimum liquid detention time of each containment system was computed, based on the effective tank volume and the estimated inflow and/or discharge rate of liquid effluent into the immediate environment. Details of these procedures for estimating or computing the flow rate and average daily volumetric discharges are provided in Appendix A-1 of the Supplementary Material.

#### 2.7.2 Sanitary sewer overflows

Sewage samples were collected from the Trichy sanitary sewers at 23 locations associated with frequent overflows. Each location was sampled at least three times during the study; once at each time interval of the day (i.e. morning between 6 and 9 a.m., afternoon between 12:00 to 3:00 p.m., evening between 4:30 to 6:00 p.m., and/or night between 7:00 p.m. and 10:00 p.m.). Samples at each site were collected through the nearest manholes. About 10 grab sub-samples of each 1-L (at an interval of 5 min) were collected from each location using either a wastewater sampler or sterilized 1-L plastic container. These sub-samples were transferred in a sterile 20-L plastic container and mixed to form a composite sample from which a 1-L aliquot was collected and taken to the laboratory for analysis. The collected samples were used to determine the pathogen load in the sewage that gets, or would get, to the surrounding environment whenever the blockages or overflows occur at the sampled locations.

According to TNUSSP (2017) and Rohilla et al. (2016a), 30% of the sewage collected in Trichy returns to the environment unsafely before reaching the treatment plant. The unsafe return per household connected to the sanitary sewer was therefore computed based on: (i) the reported daily water usage per capita in Trichy, (135 L/day), (TNUSSP, 2017); (ii) the average household size; (iii) the assumption that 80% of water used returns as wastewater; and (iv) the assumption that 30% of sewage returns to the environment unsafely. The computed volume was considered as the household average daily discharge as sewage overflow release (q_D_) in litres/day to the environment.

#### 2.7.3 Direct discharge pipes

Samples were collected from 25 household direct discharge pipes of black-water to the environment (i.e. open drains and open ground) in Trichy; none were found in NNP. Each location was sampled at least two times during the study. Samples were collected using 1000 gauge PVC plastic bags placed at the end of the selected household direct discharge pipes for about 24 h. The plastic bags were exchanged every 12 h or when full. When exchanging the plastic bags, the volume of black-water trapped in the plastic bag was measured in a sterile graduated container, and a sample collected after mixing the collected black-water. The volume of black-water collected from each direct discharge pipe was measured, and a composite sample formed using an equal volume of each of the exchanged bags (i.e. in most cases, the sub-sample volume was proportional to the collected bag volume). A 1-L sample was collected from this composite sample and taken to the laboratory for analysis. The average daily discharge as overflow (q_D_) from each direct discharge pipe (litres/day) was computed based on the collected volume of black-water discharge in a 24-h period.

### 2.8 Analytical laboratory methods

All samples were stored at below 4 °C until analysis, and these were processed within 24 h of sampling. Total solids of faecal sludge and septage samples were analysed following section 2540 B of APHA-AWWA-WEF (2017). During laboratory analysis, about 10% of the samples were analysed in duplicate and a maximum relative error of 9% was observed between the duplicates.

Samples were analysed for *E. coli* using dilution spread plate counting technique with *E. coli*-Coliforms Chromogenic Agar (Oxoid Ltd, UK), according to section 9215C of APHA-AWWA-WEF (2017). Counts were Log_10_-transformed and expressed as Log_10_ CFU per g of sample dry weight (for faecal sludge samples from lined tanks and pits) or Log_10_ CFU per ml (for liquid samples). Thereafter, *E. coli* concentrations in emptied faecal sludge or septage (C_d_) and overflows and/or effluent (C_e_) in Log_10_ *E. coli* per litre were computed.

### 2.9 Computations of *E. coli* release

Pathogens, including *E. coli,* are released from containment systems through periodic desludging as well as in liquid effluent or overflow. “Release” in this study refers to “removal from the containment system” either as “release to the environment” or “release to the next stage of the sanitation management chain”; the fate of emptied faecal sludge is often unclear, with widespread reports of clandestine dumping or use as agricultural fertilizer.

#### 2.9.1 Daily release from discharge (effluent and overflow)

The average daily *E. coli* release to the environment due to discharges (effluent and overflow) **(**$\bar{R}$_D_**)** was computed as a product of the estimated average daily overflows or effluent release (q_D_) in litres/day and *E. coli* concentrations in overflow/effluent discharge (C_D_) in *E. coli*/litre (See Eq. (2) for each system). The average daily per capita *E. coli* release **(**$\bar{R}$_D,pc_**)** was computed by dividing $\bar{R}$_D_ by the number of users of the sanitation technology (Eq. (3)).

#### 2.9.2 Daily release from desludging

The periodic *E. coli* release of desludging operations to either the environment or the “next stage of the sanitation service chain” was estimated for each containment system. During desludging of containment systems, the faecal sludge volume emptied (V_S_ in litres) from each system was estimated based on the capacity of the emptying cesspool truck (gauge scale) and/or the faecal sludge volume in the tank or pit before emptying. The total *E. coli* release from the system from a single desludging operation (R_S_) was computed as a product of the estimated volume desludged (V_S_) and the *E. coli* concentration of this faecal sludge (C_S_) (Eq. (4)). The average daily *E. coli* release from this desludging operation **(**$\bar{R}$_S_**)** was then computed by dividing R_S_ by the time T in days since the last desludging (Eq. (5)). Finally, the average daily per capita *E. coli* release due to periodic desludging ($\bar{R}$_S,pc_) was calculated by dividing R_S_ by the number of users of the system (Eq. (6)).

The average combined daily per capita *E. coli* release from sanitation technologies since last emptying ${\bar{R}}_{c,pc}$) was computed as a sum of estimated average daily per capita *E. coli* releases due to (a) discharge as effluent and/or overflow release **(** ${\bar{R}}_{D,pc}$**)**, and (b) periodic desludging operations **(**${\bar{R}}_{S,pc})$ (See Eq. (7)).

#### 2.9.3 Summary of all *E. coli* release equations

Eq. (2) $${\bar{R}}_{D}=q_{D}\times C_{D}$$

Eq. (3) $${\bar{R}}_{D,pc}=\frac{\left(q_{D}\times C_{D}\right)}{number\;of\;users}$$

Eq. (4) $$R_{S}=V_{S}\times C_{S}$$

Eq. (5) $${\bar{R}}_{S}=\;\frac{V_{S}\;X\;C_{S}}{T}$$

Eq. (6) $${\bar{R}}_{S,pc}=\left(\frac{V_{S}\;X\;C_{S}}{T}\right)/number\;of\;users$$

Eq. (7) $${\bar{R}}_{c}=\left(q_{D}\times C_{D}\right)+\left(\frac{V_{S}\;\times \;C_{S}}{T}\right)$$

Eq. (8) $${\bar{R}}_{C,pc}={\bar{R}}_{D,pc}+{\bar{R}}_{S,pc}=\left(\frac{\left(q_{D}\times C_{D}\right)}{number\;of\;users}\right)+\left[\left(\frac{V_{S}\;X\;C_{S}}{T}\right)/number\;of\;users\;)\right]$$

Where ${\bar{R}}_{D}$ *=* Average daily *E. coli* release per system due to effluent and/or overflows
${\bar{R}}_{D,pc}$*=* Average daily per capita *E. coli* release due to effluent and/or overflows
$R_{S}$*=* Average accumulated *E. coli* release per system at desludging
${\bar{R}}_{S}$*=* Average daily *E. coli* release due to periodic desludging
${\bar{R}}_{S,pc}$*=* Average daily per capita *E. coli* release due to periodic desludging
${\bar{R}}_{c}$ = Average combined daily *E. coli* release (effluent + overflow + desludging)
${\bar{R}}_{C,pc}$ = Average combined daily per capita *E. coli* release
*q*_*D*_ = Average daily discharge as overflows/effluent release in litres
*C*_*D*_ = *E. coli* concentrations in overflow/effluent discharge in *E. coli*/litre
*V*_*S*_ = Volume desludged from the containment systems in litres
*C*_*S*_ = *E. coli* concentrations in emptied faecal sludge
*T* = Time in days since the previous desludging operation

### 2.10 Statistical analysis

Household survey data were exported from mWater into Stata/SE 13.0 (College Station, Texas, USA) for cleaning and analysis. Physical measurements and laboratory data were also exported into Stata/SE 13.0. Taking the average combined daily per capita *E. coli* release of direct discharge pipes as the input or baseline, the performance of the different sanitation technologies was assessed. Data were analysed using a one-way Analysis of Variance (ANOVA) parametric test at a significance level of *p* = 0.05 to assess (a) differences amongst average daily *E. coli* release from different sanitation technologies; (b) differences between the mean values of the physical measurements (effective tank volume per capita, liquid detention time, storage periods of excreta, and emptying frequency) of the different containment system types. Average daily per capita *E. coli* release due to periodic desludging and effluent liquid discharge were compared. All significant one-way ANOVA test results were followed with a pairwise Post-hoc Tukey test, to check for the difference in means between each pair of sanitation technologies. Linear regression was used to examine the association between average daily *E. coli* release and the key design, construction, and operation features of the different sanitation technologies, with “statistical significance” determined by a >95% confidence level. Standard multiple regression analysis was conducted to identify the key design, construction, and operational features that influence performance of containment systems especially “septic tanks” (fully-lined tanks) in terms of *E. coli* release.

### 2.11 Ethical approvals

Prior to conducting user surveys, site surveys, and some key informant interviews, we obtained written informed consent from the respondents. Written consent was also obtained from the owners or operators of the sanitation systems before conducting physical measurements and environmental sampling at least 24 h in advance of the detailed survey. If owners refused or later retracted their consent, another sanitation system was randomly selected from the inventory, and the owner contacted for consent. Prior to the detailed technical study, the top slabs of the selected sanitation systems were broken to gain full access to the tank or pit and sample collection and physical measurements. However, these systems were re-sealed, and restored to their original condition or better at the end of the study. The study protocols were reviewed and approved by the Institutional Review Board (IRB) of the University of North Carolina at Chapel Hill (Reference #249163).

## 3 Results

### 3.1 Structural and physical characteristics of containment systems

The majority of the household and community containment systems in our study receive only excreta and flushing water (black-water) from the toilet facilities; only 3% of the surveyed households used containment systems as their primary means of greywater disposal; with the majority (71%) discharging greywater directly to open drains (Table S1).

All observed fully-lined tanks (household and community toilets) were rectangular. The majority (64%) of lined tanks and all lined pits were circular, with the former constructed of pre-cast concrete rings, and the latter with open-jointed pre-cast concrete rings to permit exfiltration of the liquid fraction into the surrounding soils (Table S1). Interviewees were not always aware of whether their containment were fully-lined or not; some indicated fully-lined tanks without effluent pipes where the physical survey revealed they were in fact lined.

Sixty-one percent of the observed containment systems were constructed with discharge (effluent/overflow) pipes; these included 77% of the household fully-lined tanks and 96% of the community toilet fully-lined tanks. All effluent/overflow pipes discharged to open drains (72%); stream, pond, or river (5%); open ground (12%), and soakaways or drain fields (11%) (Fig. 1 and Table S1). We frequently observed fresh faeces in the effluent from community toilet fully-lined tanks to the environment. All lined tanks and lined pits had no discharge pipes, and their pathogen release is entirely through exfiltration and desludging. Note that we never observed any exfiltrated excreta/liquid on the surface within the household or community environment during field investigation. Containment system characteristics are shown in Table S1.

### 3.2 Design and operational characteristics of the containment systems

The main design and operational characteristics of the studied containment systems were computed and compared, to permit study of their effect upon performance in *E. coli* removal (Table 3). The effective tank volume per capita varied significantly by technology. Community toilet fully-lined tanks had the lowest effective tank volume per capita, less than 10% of that for the fully-lined household tanks; these differences were statistically significant at p < 0.0001 (Table S4 which includes the pairwise Post-hoc Tukey test results). The mean liquid detention time of household fully-lined tanks (15.6 h) was significantly different to that of community toilet fully-lined tanks (5.0 h) (*p* = 0.0001).

**Table 3:** Key design and performance characteristics of studied containment systems.

Type of the containment systems	178 Containment Systems studied				
	Household Systems				Community Toilets
	Fully-Lined Tank with effluent pipes (n = 81)	Fully-Lined Tank without effluent pipes (n = 24)	Lined Tanks (n = 24)	Lined Pits (n = 19)	Fully-Lined Tank (n = 28)
**Characteristics and Design Features**	Median or Mean ± SD; Range (Min - Max)	Median or Mean ± SD; Range (Min - Max)	Median or Mean ± SD; Range (Min - Max)	Median or Mean ± SD; Range (Min - Max)	Median or Mean ± SD; Range (Min - Max)
Number of users	8 ± 6 (2–35)	8 ± 5 (3–20)	6 ± 3 (2–15)	5 ± 2 (3–8)	204 ± 129 (30–500)
Effective Tank Volume per capita (litres/user)	880^a^ (126–3,689)	952^a^(214–3,964)	245^a^ (40–2,170)	191^a^ (40–640)	84^a^ (14–510)
Liquid Detention time (hours)	15.6 ± 11.8 (3.1–68.6)	N/A	N/A	N/A	5.0 ± 3.1 (1.3–15.1)
Storage periods between emptying (months)	24.3^a^ (2.9–61.6)	11.1^a^ (0.8–29.4)	21.2^a^ (0.03–313)	57.1^a^ (12.3–178.8)	0.9^a^ (0.03–313.4)
Emptying Frequency in the past 5 year	3^a^ (1–15)	7^a^ (2–120)	5^a^ (1–30)	1^a^ (1–5)	60^a^ (1–1800)

a Median, Min = Minimum, Max = Maximum, and SD=Standard deviation. Data collected on some variables were unevenly distributed and very highly variable, therefore, median instead of mean values are reported, denoted with *.

Lined pits were associated with the longest storage periods of excreta between desludging of about 57 months; followed by household fully-lined tanks with effluent pipes (24 months), lined tanks (21 months), fully-lined tanks without effluent pipes (11 months), and community toilet fully-lined tanks (about 1 month).

The emptying frequency in the past 5 years differed significantly across the observed containment system (*p* < 0.0001, See Table S4 - for details). The majority of the household fully-lined tanks with effluent pipes, fully-lined tanks without effluent pipes and lined tanks were emptied about 2–10 times in the past 5 years. In contrast, community toilet fully-lined tanks were emptied >60 times, and lined pits <2 times in the same period (Table S3).

### 3.3 *E. coli* release estimation from sanitation systems

#### 3.3.1 *E. coli* release due to liquid discharge

**Fig. 2:**
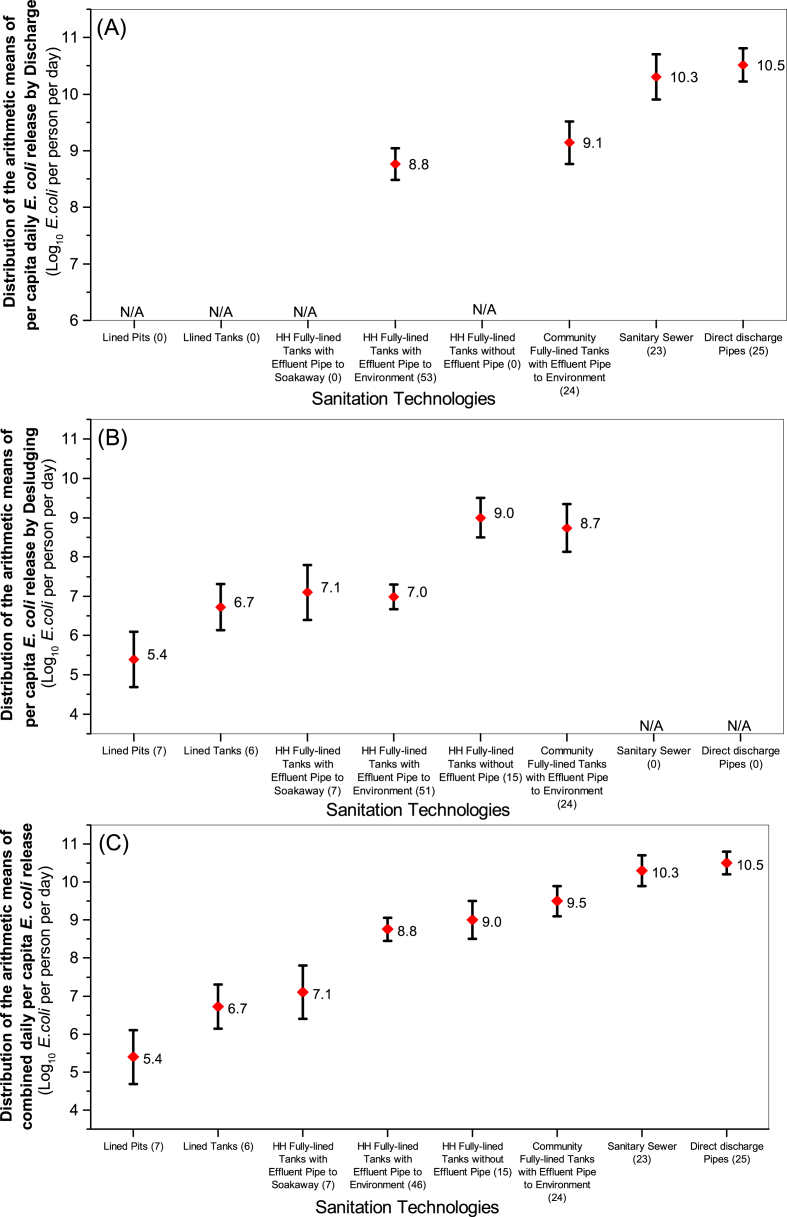
(A) Arithmetic mean of the average daily per capita *E. coli* release from sanitation technologies to the environment due to discharge (i.e. effluent and/or overflows); (B) Arithmetic mean of the average daily per capita *E. coli* release to the next stage of the sanitation service chain and/or environment due to period desludging; (C) Arithmetic Mean of the average combined daily per capita *E. coli* release to the next stage of the sanitation service chain and/or the environment. *Error bars represent 95% confidence limits of the geometric means*.

Fig. 2 (A) presents the arithmetic means of the average daily per capita *E. coli* release to the environment due to liquid discharge from the different sanitation technologies with effluent or overflows. Overflows from sanitary sewer points and direct discharge pipes exhibited the highest per capita daily *E. coli* release, with the arithmetic means of 10.3 and 10.5 Log_10_ *E. coli* per person per day, respectively. Household fully-lined tanks with effluent pipes to the environment exhibited the lowest average liquid daily *E. coli* release per capita (8.8) followed by community toilet fully-lined tank, (9.1) Log_10_ *E. coli* per person per day. There are significant differences in the daily per capita *E. coli* releases by discharges from different sanitation technologies, with *p* = 0.0001. Similarly, the pairwise Post-hoc test with a Tukey adjustment revealed significant differences in *E. coli* release between each pair of sanitation technologies (with *p* = 0.0001), with exceptions of (i) direct discharge pipes and sanitary sewers (*p* = 0.9), and (ii) household fully-lined tanks with effluent pipes to environment and community fully-lined tanks (*p* = 0.4) (Table S5).

#### 3.3.2 *E. coli* release due to periodic desludging

Fig. 2 (B) presents the arithmetic means of the average daily per capita *E. coli* releases due to periodic desludging of the different technologies. Lined pits recorded the lowest average daily per capita *E. coli* release to the environment (or next stage of the sanitation service chain) due to periodic desludging, with the arithmetic mean of 5.4 Log_10_ *E. coli* per person per day; followed by lined tanks (6.7), household fully-lined tanks with effluent pipes (7.1), community toilet fully-lined tanks (8.7) and finally household fully-lined tanks without effluent pipes (9.0). Based on the pairwise post-hoc tests with a Tukey adjustment, the difference in average daily *E. coli* release due to periodic desludging between each pair of sanitation technologies was highly significant with *p* < 0.05, with the exceptions of (i) household fully-lined tanks with effluent pipes to the environment and those with soakaways, (ii) lined tanks and household fully-lined tanks with effluent pipes to environment, (iii) lined tanks and household fully-lined tanks with effluent pipes to soakaways, (iv) lined pits and household fully-lined tanks with effluent pipes to soakaways, (v) household fully-lined tanks without effluent pipes and community toilet fully-lined tanks, (vi) lined pits and lined tanks (Table S5).

#### 3.3.3 Combined *E. coli* release from liquid discharge and desludging

**Fig. 3:**
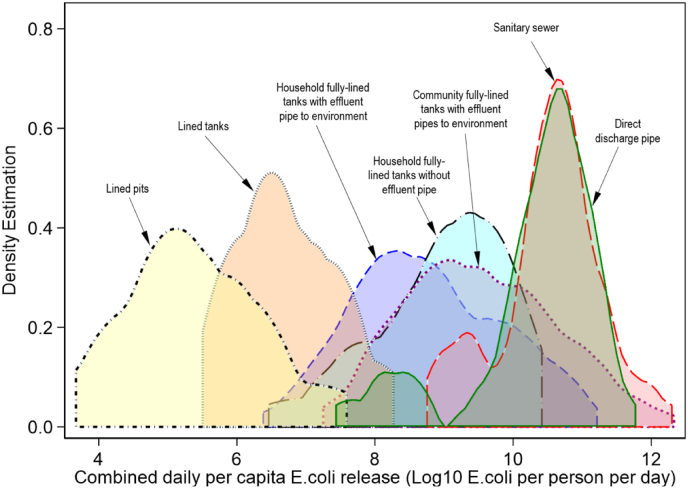
Distribution of the average combined daily per capita *E. coli* release from the different sanitation technologies to the environment and/or next stage of the sanitation service chain. Density estimation represents the probability density function of the normalised variable (i.e. average combined daily per capita *E. coli* release across the population of each sanitation technology). This density plot displays the distribution of data over a continuous interval of combined daily per capita *E. coli* release. The peaks of a Density Plot for each sanitation technology shows where values are concentrated over the interval of average combined daily per capita *E. coli* release.

For each individual system, the average daily per capita *E. coli* release from liquid discharge was arithmetically summed with the average daily per capita *E. coli* release from periodic desludging to compute the average combined daily per capita *E. coli* release. Fig. 2 (C) thus shows the arithmetic means of the average combined daily per capita *E. coli* release to the environment and/or the next stage of the sanitation service chain. This statistic differed significantly across the sampled sanitation technologies, with *p* = 0.00001 (See Fig. 3). The lined pits, lined tanks and household fully-lined tanks with effluent pipes to soakaways exhibited the lowest arithmetic average combined daily per capita *E. coli* release of about 5.4, 6.7 and 7.0 Log_10_ *E. coli* per person per day, respectively, as shown in Fig. 2 (C). Unsurprisingly, direct discharge pipes recorded the highest average combined daily *E. coli* release, with 10.5 Log_10_ *E. coli* per person per day; as shown in Fig. 2(C), these were followed by sanitary sewers (10.3), community toilet fully-lined tanks (9.5), household fully-lined tanks without effluent pipes (9.0), household fully-lined tanks with effluent pipes to the environment (8.8). The pairwise post-hoc tests with a Tukey adjustment further confirmed that the difference in average combined daily per capita *E. coli* release between each pair of sanitation technologies was significant with *p* < 0.05 but with the following exceptions: (i) household fully-lined tanks without effluent pipes and those with effluent pipes to the environment, (ii) household fully-lined tanks without effluent pipes and community toilet fully-lined tanks, (iii) lined tanks and household fully-lined tanks with effluent pipes to soakaways, (iv) lined pits and lined tanks, (v) sanitary sewer overflows and community toilet fully-lined tanks, and (vi) sanitary sewers and direct discharge pipes (Table S5).

### 3.4 Containment system performance in reducing *E. coli* release

We have termed household systems which discharge the deposited excreta directly to the environment with no storage or treatment as “direct discharge pipe systems”. Taking the average combined daily per capita *E. coli* release of these systems as the baseline or input, we can compare the relative effectiveness of other technologies in reducing *E. coli* before their release. Lined tanks, lined pits and household fully-lined tanks with effluent pipes to soakaways exhibited excellent performance with approximately 3.5–5.1 Log_10_ *E. coli* per capita reduction, followed by household fully-lined tanks with effluent pipes to the environment (1.7), household fully-lined tanks without effluent pipes (1.5), community toilet fully-lined tanks (1.0), and sanitary sewer overflows (0.2) (See Fig. 2 (C)).

## 4 Discussion

### 4.1 Design and operational characteristics of on-site sanitation technologies

The design, structural and operational characteristics of containment systems such as effective tank volume, effective tank volume per capita, liquid detention time, storage periods of excreta prior to desludging, effluent disposal, and emptying frequency varied significantly between the different sanitation technologies (Table S4).

#### 4.1.1 Community fully-lined tanks vs household fully-lined tanks

The storage volume/user in the community toilet fully-lined tanks is less than a tenth of that for the household units (See Table 3). This greater storage volume also implies that, depending on the daily wastewater generation rate per capita in the study communities, the household fully-lined tanks would be associated with longer hydraulic detention times (of about 15 h) than community toilet fully-lined tanks (of about 5 h). This is the reason for considering “household fully-lined tanks” and “community fully-lined tanks” as distinct technologies. The average dentition time of household fully-lined tanks observed in our study is within the threshold detention time range (of 12–24 h) that is acceptable for removal of total solids in “septic systems” (Nnaji and Agunwamba, 2012).

#### 4.1.2 Lined pits vs lined tanks

Our study revealed that some of the key design and operational features (such as effective tank volume per capita, storage periods of excreta before emptying, etc.) of the lined pit and lined tanks were not significantly different (Tables S1–S4), suggesting that these containment systems are similar in their design and functionality. However, the lined tanks had to be emptied nearly three times as often as the lined pits. This may be because the lined tanks are associated with limited liquid exfiltration through the tank bottom only, while the lined pits are designed and constructed to allow exfiltration of the liquid fraction both through the bottom and semi-permeable sidewalls of the pit into the surrounding soils. We also observed a difference in the characteristics of faecal sludge collected from lined pits and lined tanks – as the faecal sludge from the former was more viscous and contained more solids content (with about 11.2% total solids) than that from the latter (with about 6.9% total solids). This may be attributable to (a) possible differences in influent material, with less water being flushed into pits than tanks, and (b) the corresponding difference in the storage periods of faecal sludge between desludging - as previous research has shown the total solids of faecal sludge from the containment systems (especially lined pits) to increase with the increase in storage times between emptying (Englund et al., 2020).

#### 4.1.3 Number of users, detention time, and emptying frequency

Community toilet fully-lined tanks were associated with both the shortest storage periods between emptying and the highest emptying frequency over the previous 5 years (Table 2). This is logically due to the high user rate (about 170 people per community toilet fully-lined tank compared to 6–7 people for other lined tanks), combined with smaller effective tank volume per capita of the community toilet fully-lined tanks – all resulting from the under-design of the community toilet containment systems, with reference to recommended hydraulic detention time (Bounds, 1997; Bureau of Indian Standards, 1993; USEPA, 2002; Brandes, 1978). A similar observation was reported in Englund et al. (2020) study that found the emptying frequency of “septic tanks” to increase with increase in the number of users and decrease in the volume of the containment system. The short storage periods between desludging of community toilet fully-lined tanks observed in this study are comparable to those reported in literature, for example, Strande et al. (2018) similarly found community toilet “septic tanks” to be associated with high emptying frequency (of about 1825 in 5 years) and very short storage periods between emptying of as short as one day.

Lined tanks and pits have longer solids storage and lower emptying frequency than other technologies, perhaps because some of the liquid fraction of the biodegrading excreta exfiltrates into the surrounding soils, while the solids accumulate in containment more slowly. In the same vein, household fully-lined tanks without effluent pipes were associated with shorter storage periods between emptying and thus higher emptying frequency, than those fully-lined tanks with effluent pipes (See Table 2); this reflects the fact that fully-lined tanks without effluent pipes are constructed to store both the liquid and solid fraction of excreta, while those with effluent pipes are designed and built to release the liquid fraction to either the environment or soakaways while the solid fraction slowly accumulates in the tank.

### 4.2 *E. coli* concentrations and average daily volumetric discharges

The arithmetic mean of *E. coli* concentrations we found in the liquid discharges and faecal sludge/septage from the different sanitation technologies were in the range of 7.0–9.0 Log_10_ and 5.5–8.2 Log_10_ *E. coli*/L, respectively, and 9.0 Log_10_ *E. coli*/L for direct discharge black-water. (Table S8). Our findings broadly align with the published *E. coli* concentrations in faecal sludge/septage (Bassan et al., 2013; Manga et al., 2016; Manga, 2017) or liquid effluent (Abbassi et al., 2018; Amin et al., 2020; Pang et al., 2004; Richards et al., 2016; Humphrey et al., 2011; Harwood et al., 2017) from sanitation technologies, and direct discharge black-water (Mawioo et al., 2016; Eregno et al., 2018). However, no study to our knowledge has systematically studied concentrations of *E. coli* in both the liquid discharges and faecal sludge/septage from on-site sanitation technologies.

Table S8 presents the arithmetic means of the average daily per capita liquid discharges and faecal sludge/septage volumes from the different sanitation technologies, and these were in the range of 20–69 L per capita per day. Currently, we are unaware of any published studies which reports volume estimates of liquid and faecal sludge discharges from sanitation technologies.

### 4.3 *E. coli* release by liquid discharge (overflows and/or effluents)

Our study found that direct discharge pipes and sanitary sewer overflows released a higher average daily per capita *E. coli* load in liquid discharge to the environment than on-site containment systems (e.g. household and community toilet fully-lined tanks); recall that lined pits and tanks in our study areas have no effluent or overflows. The results indicate that shifting from direct discharge pipes to on-site sanitation technologies with containment systems (especially those with the added advantage of liquid fraction exfiltration) may result in a 1–2 Log_10_ reduction in the average daily per capita *E. coli* release to the environment due to liquid discharge. This is in alignment with the well-established fact that some partial treatment of excreta and inactivation or removal of *E. coli* occurs during safe containment of excreta (Feachem et al., 1981; Franceys et al., 1992; Mara, 1996). Other studies (Odagiri et al., 2021; Maxcy-Brown et al., 2021) have also found that the direct discharge of black-water to the environment is a common practice in India and elsewhere, which we believe results in the substantial release of faecal pathogens to the environment, and a substantive public health hazard. We observe from our data in Fig. 2 that direct discharge pipes and sanitary sewer overflows (without treatment) may be viewed as “hypodermic needles” of pathogens, injecting up to 1000 times more *E. coli*/person/day than the other on-site sanitation systems we observed.

The volumetric liquid discharges (litres per capita per day) from household fully-lined tanks with effluent pipes to the environment were about 3 times *higher* than those from community toilet fully-lined tanks (Table S8). However, the daily per capita *E. coli* discharge from these household systems were about 3 times (0.4 Log_10_ *E. coli*) *lower* than these community toilets. This may be due to the longer liquid detention times in household tanks (of about 15.6 h), relative to community systems (5.0 h). Previous studies have similarly shown that septic tanks with longer hydraulic detention time perform better in terms of both solids and pathogen removal from tank effluent (Bounds, 1997; Nasr and Mikhaeil, 2013; Koottatep et al., 2004; Nnaji and Agunwamba, 2012). In this study, we observed evidence that an increase in liquid detention time of the containment system by 1 day was associated with 0.7 Log_10_ reduction in the average daily per capita *E. coli* release due to liquid discharge (R^2^ = 0.11, *p* = 0.003, see Table S7). In the same vein, the high *E. coli* release associated with the community toilet fully-lined tanks can be attributed to hydraulic overload due to the high usage rate of the community toilet fully-lined tanks and small effective tank size per capita, which all result in shorter and inadequate liquid detention times.

Observed discharge concentrations from fully-lined tanks with longer hydraulic detention times were lower than those with shorter hydraulic detention times. This is consistent with previous studies that found longer liquid detention times permit better removal of settleable solids and associated pathogens from the liquid discharge through sedimentation (Brandes, 1978; Nnaji and Agunwamba, 2012).

### 4.4 Release of *E. coli* from periodic desludging

Community toilet fully-lined tanks and household fully-lined tanks without effluent pipes showed the highest average daily per capita *E. coli* release from desludging (See Fig. 2 (B)) than other containment systems. This may be attributed to the short storage of excreta before desludging associated with community toilets and household fully-lined tanks without effluent pipes. This observation was supported by a meaningful association between the storage periods of excreta and average daily per capita *E. coli* release due to periodic desludging (R^2^ = 0.21, *p* = 0.00001). Further, we observed evidence that an increase in the emptying frequency of the containment system was significantly associated with a higher average daily per capita *E. coli* release due to periodic desludging (R^2^ = 0.16, *p* = 0.00001).

Our study results suggest that excreta safely contained for an extended storage period in lined tanks and pits before desludging, contains less pathogen indicator organisms and thus presents less of a public health hazard than fresher excreta from community toilet fully-lined tanks. A similar observation was reported by Mills et al. (2018) who indicated that reducing the emptying frequency and extending the storage periods of human excreta has a potential of reducing the public health risks - as no exposures are assumed to be associated with safely contained and unemptied faecal sludge. In the same vein, previous researchers have similarly found safe containment of excreta for longer periods to be responsible for pathogen reduction (Feachem et al., 1983). The difference in the average daily *E. coli* release due to periodic desludging between the lined tanks and household fully-lined tanks with effluent pipes was not significant (*p* = 0.994) implying similar performance of these two types of tanks when it comes to the containment of the solid fraction of excreta or septage. This evidence was also confirmed by the similarity in storage periods of both types which were in the range of 21–24 months (see Table 3).

### 4.5 Effectiveness of sanitation technologies in reducing *E. coli* release

In this study, we have focused on pathogen release routes that were accessible to us: liquid discharge, and desludging. We cannot, from our study, know what fraction of desludging is safely managed, as we were not able to trace the fate of such faecal sludge from emptying to its final return to the environment. Our work, however, enables one to assess the relative benefits of improved management of liquid discharges, versus better offsite treatment of faecal sludge and its safe disposal. In the following discussion, we have tentatively assumed, for demonstrative purposes, that all the pathogens still present in faecal sludge at emptying are unsafely returned to the environment, to assess the degree of indicator organism *E. coli* removed by on-site containment and treatment, by the different technologies; the implications of this assumption are explored later.

Overall, the performance of the different sanitation technologies varied significantly (Table S5). This can be attributed to the principal routes through which the different sanitation technologies release *E. coli* to the environment or the next stage of the sanitation service chain. For example, in our study, the lined tanks, lined pits, and household fully-lined tanks with effluent pipes to soakaways, primarily released *E. coli* through a single route of periodic desludging after safe containment of excreta for long periods of about 21–57 months (see Table 3) as the liquid fraction exfiltrates through the sidewalls and/or bottom of the tanks/pits. Similarly, the household fully-lined tanks without effluent pipes release *E. coli* through a single route of periodic desludging, but only after safe containment of both the liquid and solids fraction of excreta for a short time of about 11 months (see Table 3). However, community toilet fully-lined tanks and household fully-lined tanks with effluent pipe to the environment released *E. coli* through both periodic desludging after storage periods of about 1–24 months, and frequent release of effluents. Currently, we have found no study in the literature reporting comparable data.

#### 4.5.1 Benefits of soakaways

Previous studies have reported discharge of containment effluent to the environment as a common practice in India, with about 72% of containment systems discharging effluent to open drains, especially in the urban environment (Dasgupta et al., 2019). This study and other studies in Dhaka, Bangladesh (Amin et al., 2019) and Indonesia (Odagiri et al., 2021) have found this practice to contribute to the release of faecal pathogens to the environment, constituting a clear public and environmental health hazard. However, our findings suggest that, where feasible, changing from the household fully-lined tanks with effluent pipes to the environment to effectively the same system discharging to soakaways would yield a 1.8 Log_10_ reduction in the average combined daily per capita *E. coli* release. Similarly, switching from household fully-lined tanks with no effluent pipes, (in which case septage is simply stored until emptying) to the same system with a soakaway, can reduce the per capita *E. coli* release by 2 orders of magnitude. Fully-lined tanks with effluent pipes connected to soakaways pose less risks of public exposure to faecal pathogens than equivalent systems discharging their liquid content directly to the environment. For this reason, our findings are consistent with previous analyses of sanitation technologies that concluded that all “septic systems” should comprise of a drainfield or soakaway for exfiltration of the liquid effluent into the surrounding soils for further pathogen inactivation or removal (Foster et al., 2021). In the same vein, connection of fully-lined tanks effluent to soakaways reduces the faecal pathogens in the liquid fraction in the environment, which subsequently reduce exposure risks at the household and community open drains (Mills et al., 2018).

#### 4.5.2 Relative magnitudes of *E. coli* release from effluent and sludge

In the case of household fully-lined tanks with effluent pipes to the environment*,* we found the daily per capita *E. coli* release associated with the liquid fraction (effluent discharge) is approximately 63 times higher than from faecal sludge emptying. The ratio of liquid/solid per capita loadings of *E. coli* is reduced to 2.5 in the case of community toilet fully-lined tanks. Importantly, these ratios of liquid effluent/faecal sludge loadings of *E. coli* “discharge to the environment” would be *higher* if the collected faecal sludge is in fact properly managed; we draw the key conclusion that whether or not the faecal sludge management chain is effective, the *E. coli* loading to the environment from these systems is predominantly in the liquid effluent. This is a significant finding for sanitation planners and engineers as it may well aid the prioritizing of sanitation interventions that will minimize release of pathogen indicator organism *E.coli* to the environment and minimize e public health hazard. Our study findings are in agreement with Peal et al. (2020) that concluded that safely managed sanitation cannot be achieve by managing only faecal sludge but also the liquid effluent – as this will help reduce the release of pathogen hazards to the environment.

#### 4.5.3 Ranking of technologies by *E. coli* release

These study findings are consistent with the observation made by Kolsky et al. (2019) that from a perspective of pathogen hazards release, the widely-held public view of a sanitation technology hierarchy of “safety” from pit latrines → septic tanks→ sanitary sewers, is probably incorrect as such systems are currently managed in resource-poor settings. Arguably, our data suggest that in many settings it may be reversed, and the hierarchy should start with the sanitary sewers with inadequate sewage treatment before discharge as the worst performance, lined pits with the best performance, and the other technologies in-between may be ranked on the basis of the per capita *E. coli* loadings to the environment.

#### 4.5.4 Effects of excreta storage, liquid discharge, and faecal sludge management

The excellent performance exhibited by the lined tanks and lined pits can be attributed to (a) the long excreta storage periods prior to desludging and low emptying frequency associated with such containment systems, and (b) the lack of liquid discharge to the environment. An increase in the storage periods of excreta and a decrease in the emptying frequency was significantly associated with a decrease in the average daily per capita *E. coli* release, thus performance (Tables S6–S7). Even though the lined tanks and lined pits exhibited excellent performance, these systems still release 5.4–6.7 Log_10_ *E. coli* per person per day to the environment or next stage of the sanitation service chain; safer management of excreta along the service chain is still needed to minimize the unsafe returns and the associated pathogen hazards. Our findings are consistent with previous analyses on pathogen flows associated with sanitation technologies that if on-site sanitation technologies are to adequately protect public health and contamination of the wider environment, they all require appropriate and robust management regardless of their performance in terms of inactivating or removing faecal pathogens (Foster et al., 2021).

Amongst the containment systems, community toilet fully-lined tanks exhibited the worst performance, with the highest average daily per capita *E. coli* release. This can be attributed to the short liquid detention time, short storage periods of the excreta prior to emptying, and high emptying frequency associated with community toilet fully-lined tanks from hydraulic overloading or higher user rate, and smaller effective tank volume per capita. This observation was supported by a meaningful correlation between average daily per capita *E. coli* release and storage periods of excreta before desludging (R^2^ = 0.16, *p* = 0.0006), liquid detention time (R^2^ = 0.13, *p* = 0.0025) and emptying frequency (R^2^ = 0.21, *p* = 0.0001).

Our results confirm that safe containment of excreta for an extended period, with resultant pathogen inactivation, has great potential to reduce the release of pathogen hazards to the next stage of the sanitation service and/or environment as well as the spread of excreta-related diseases. However, both the rate of pathogen inactivation and the factors influencing pathogen reduction or inactivation in containment systems vary widely between pathogens, and are still not well understood.

Our observation of no statistically significant difference between the performance of community toilet fully-lined tanks and sanitary sewer overflows (with *p* = 0.110), suggests that such community toilet fully-lined tanks probably function more like direct discharge pipes. The poor performance exhibited by community toilet fully-lined tanks could reflect both the short liquid detention time and the poor operation and emptying practices of these systems. By visual observation of the emptying operations, most of the these tanks were partially emptied especially from the second or third chamber of the tank; this could be due to the large sizes of the containment system, where the desludging operators could not completely desludge the tank in a single trip. Failure to empty the first chamber naturally leads to continuous accumulation of faecal sludge in this tank over several cleanings, thus gradually reducing the effective tank volume. Similar observation was made by Koottatep et al. (2014) and Nnaji and Agunwamba (2012) that the accumulation of faecal sludge in the containment system reduces the mean hydraulic detention times, which results in more short-circuiting and an increase in the pathogens and pollutant release from the containment system.

### 4.6 Design and operational features influencing performance

Regression results revealed safe disposal of the liquid effluent to soakaways as the most important design feature that reduces *E. coli* release from “septic systems”, as this effectively eliminates the liquid release route. We observed that construction of the fully-lined tank with liquid effluent disposal to soakaways significantly reduces average daily combined per capita *E. coli* release by about 30 times (Coef. = −0.903, *p* = 0.029), and this was the highest reduction observed in the study. This was followed by the inlet and outlet pipe configurations (Coef. = −0.892, *p* = 0.0001), liquid detention time of ≥24 h (Coef. = −0.563, *p* = 0.011) and faecal sludge storage times between desludging (Coef. = −0.180, *p* = 0.026). These findings suggest some of the important design features and operational practices that should be considered for any “septic system”/fully-lined tank, from the perspective of reducing pathogen releases. Soakaways, however, may not be technically or financially realistic in areas with rock or tight soils; in this case, both inlet and outlet pipe configurations and detention times take on greater importance. Previous studies, have also found proper configuration of inlet and outlet pipes (Koottatep et al., 2014) and hydraulic detention time (Brandes, 1978; Nnaji and Agunwamba, 2012) as important features to consider during the design and construction of fully-lined tanks so as to improve the performance of the systems to better safeguard public health and protect environmental contamination.

Further, although some studies and design manuals (Koottatep et al., 2014; Georgia Department of Public Health, 2019; Missouri DHSS., 2018) have recommend other key design features of “septic systems” or fully-lined tanks (such as Length: Breadth ratio of ≥1.5, number of chambers, effective tank depth of 0.9–2, etc.), in our study we did not, however, observe a meaningful association between them and the performance indicator of average daily *E. coli* release (See Supplementary information; Table S7). This may be because our sample size was insufficient to detect the effect of these design features on the *E. coli* release, as some of these design features were missing in the majority of the sampled fully-lined tanks. However, our study still encourages us that there are many plausible ways to improve performance, and that rigorous studies to test these design features and their relative impacts would be desirable.

Inlet and outlet pipe configuration had a statistically significant association with average daily *E. coli* release due to average daily combined per capita *E. coli* release. Our results suggest that use of correct inlet and outlet pipe configuration reduces the average daily combined per capita *E. coli* release by about 8 times. Liquid detention time had a statistically significant association with the average daily combined per capita *E. coli* release. An increase in liquid detention time by 1 day reduces average daily *E. coli* release due to the average daily combined per capita *E. coli* release by about 7 times (See Table S7). Therefore, the proper designing of septic tanks/fully-lined tanks with a minimum liquid detention time of ≥1 days should be emphasised.

There is a practically meaningful and statistically significant relationship between storage periods of excreta before desludging and average daily per capita *E. coli* release due to average daily combined per capita *E. coli* release. We observed that safe containment of excreta for prolonged periods significantly reduces *E. coli* release to the environment or next stage of the sanitation service chain. This implies that the containment system should be designed with a sufficient effective tank volume per capita for storage of the excreta to promote adequate pathogen inactivation or removal during containment. Sanitation technologies such as lined tanks and lined pits with storage periods of more than 21 months were associated with considerably lower *E. coli* release due to periodic desludging. Further studies are needed to better understand the die-off kinetics of pathogens in the septic tank sludge and timing of the pseudo-steady state of pathogen load in the tanks.

### 4.7 Limitations of the study

We believe that both the results of this study, and the approach taken of tracking pathogen flow through both effluent and faecal sludge disposal routes may be of significant value to those working in this area. Like all studies, however, this one has some limitations.

Due to logistical challenges, the sanitation systems studied were limited to those we encountered during the community visits and transect walks; all the systems in the final study were purposively selected to represent a range of sanitation technologies observed in the study communities. A fully representative sample was not possible. This means we cannot, with scientific rigour, generalize some of the key findings and conclusions of this study to the study area population, let alone to all the communities in Tamil Nadu or other cities in India. However*,* the study findings should be helpful for policymakers and sanitation planners in considering similar study communities or contexts, and to promote better priority setting and decision-making in sanitation interventions.

In broad terms of public health importance, we are at best estimating *E. coli* release to the environment, not public exposure to these *E. coli*; details of where such releases went and their possible impact on human exposure were beyond the scope of the study. The authors nevertheless believe that one of the first steps for better sanitation management for public health is an understanding of the pathogen flow and “leakage” from the systems designed to control excreta.

In our study, periodic desludging and liquid discharge (overflow or effluent release) were considered as the principal routes for pathogen (represented in this study by *E. coli*) release from sanitation technologies to the environment. We did not consider other pathogen release routes such as exfiltration of the liquid fraction from the lined tanks and lined pits, or the soakaways for fully-lined tanks. During our observations, we did not observe and were unaware of any surfacing of the exfiltrated excreta/liquid, but this could be much more significant during rains. The associated risk from exfiltration into soil is, of course, heavily dependent upon the location of any groundwater sources used for drinking water.

Secondly, the unsafe returns and *E. coli* releases associated with emptying, transportation, treatment, and disposal were not considered in this study as we focused on the release from the sanitation technologies or containment systems. However, our related work in progress on pathogen flow addresses pathogen releases from other stages of the sanitation service chain.

Like many before us, we used *E. coli* as an indicator for pathogens released from the different sanitation systems because it is has been widely reported in literature as an important and very widely-used faecal indicator when assessing the microbial and public health hazards and/or risks associated with faecal waste/human excreta (Feachem et al., 1983; Odonkor and Ampofo, 2013; Manga et al., 2021). However, *E. coli* may not be a suitable organism indicator for some pathogens (such as helminth eggs, viruses, protozoa oocysts etc.) found in faecal sludge/human waste from different sanitation technologies (Amin et al., 2019).

## 5 Conclusions

While there is a wide range of on-site sanitation technologies used in much of the world, there has been little systematic study of their relative effectiveness in reducing pathogen release in either liquid or solid waste streams, or of the combined release of pathogens to the environment from these two streams. Field investigations in Tamil Nadu, India were undertaken to assess the performance of local sanitation technologies in reducing the release of the indicator organism *E. coli* to either the environment or the next downstream step of the sanitation service chain. -We hope these findings, (combined with future work by this team and others adopting a similar approach with different pathogens, and in other sites), will be useful in improving the design, construction and operation, and performance of on-site sanitation technologies (and especially septic systems) in terms of pathogen inactivation and release. Based on the study findings, we draw the following conclusions:

i. Pathogen release from the studied on-site sanitation technologies varied by as much as 5 orders of magnitude from “lined pits” (5.4 Log10 *E. coli* per person per day) to “overflowing sanitary sewers” and “direct discharge pipes” (10.3–10.5 Log10 *E. coli* per person per day). Other technologies (“lined tanks”, “household fully-lined tanks”, and “community toilet fully-lined tanks”) lay between these extremes, and their performances in *E. coli* removal also varied significantly, in both statistical and practical terms. “Household fully-lined tanks”, a widespread technology, offered nearly a 2 Log10 reduction in *E. coli* compared to “direct discharge pipes” in which no detention or treatment occurs.
ii. Human excreta safely contained for an extended storage period in lined tanks and lined pits contained significantly less *E. coli* than fresh excreta discharged from direct discharge pipes or sanitary sewers without treatment. This study indicated that the direct discharge pipes and sanitary sewer overflows (without treatment) release up to 1000 times more *E. coli*/person/day to the environment than the on-site sanitation systems we observed. Therefore, sanitary sewer authorities and funders should not just prioritize the extension of sanitary sewer services but should also stress (i) improved operations and maintenance to reduce sanitary sewer blockages and overflows to minimize highly concentrated pathogen release, and (ii) effective and reliable pathogen removal at treatment works.
iii. Community toilet fully-lined tanks exhibited higher *E. coli* release per capita than household fully-lined tanks, and this was significantly influenced by the mode of operation. Therefore, sanitation engineers and authorities should pay more attention to improvement to the operation, routine maintenance of similar systems to improve performance.
iv. Community toilets and household “septic tanks” (the most commonly used technology in the study area) at present discharge on average 3 and 65 times, respectively, as many *E. coli* per person per day through the daily liquid release than through periodic desludging. Our study findings suggest that although faecal sludge management along the sanitation service chain is important, the highest priority should be directed to proper management of the liquid effluents from these containment systems. The significance of liquid stream release from “septic tanks” and other on-site sanitation technologies is also highlighted by the 2–3 Log10 difference between systems without significant liquid release (lined pits, lined tanks and fully-lined tanks with effluent pipes to soakaways), and those with significant liquid release (fully-lined tanks with effluent pipes to environment). The former exfiltrate their liquid fraction through soakaways or the soil, without creating, in the sites we studied, any visible threat or nuisance, while the latter release most of their *E. coli* to the surface as effluent or overflow.
v. Four design parameters were found to influence performance of on-site sanitation systems in *E. coli* removal: (i) disposal of the liquid effluent to soakaways, (ii) inlet and outlet pipe design to reduce short-circuiting, (iv) liquid detention time and (iii) faecal sludge storage time to reduce the frequency of emptying, as it affected the *E. coli* released. The study suggests that to minimize average daily *E. coli* release associated with the liquid fraction of excreta, the plumbing errors in the inlet and outlet pipe configurations need to be addressed as soon as possible, as they lead to serious “short-circuiting”. Further, proper design of the containment system is important – to improve the performance in terms of reducing pathogen indicator organism *E. coli.* However, proper operation and maintenance practices of the containment systems are at least as important as technology selection, design, and construction.

Future work should focus on i) detailed analyses of technical and process factors in performance, including a rigorous comparison of what are known locally as “septic tanks” with the key criteria adopted nearly universally by engineers and boards of health in defining an acceptable septic tank; and ii) development of a “pathogen flow diagram” for the communities to highlight where in the sanitation service chain the greatest pathogen leaks to the environment occur.

## Disclaimer

The findings and conclusions of this study are solely of the authors, and do not represent the views, decisions or policies of the institutions with which they are affiliated.

## Author contribution

Conceptualization (MM, PK, JS, JB, & JR); Methodology (MM, PK, JS, & JR); Software (MM); Formal Analysis (MM, & PK); Investigation (MM, PK, & JS); Resources (PK, JB, & JS); Data curation (MM, PK, JS, JB, & JR); Writing - original draft preparation (MM); Writing – Review and Editing (MM, PK, JB, JS, JR, SR, & LS); Visualization (MM, PK); Supervision (JS, PK, & JB); Project Administration (MM, JS & PK); Funding Acquisition (PK, JS & JB). All authors read and approved the final manuscript.

## Funding

This study was financially supported by the 10.13039/100000865Bill and Melinda Gates Foundation, Grant No OPP1158911.

## Footnotes

1 A ‘transect walk’ is a systematic walk by researchers and community members along a defined path (transect) across the community/project area to explore the sanitation conditions by observing, asking, listening, looking and producing a transect diagram (World Bank, 2016; Keller, 2020).
