# Supplementary material for: Public health performance of sanitation technologies in Tamil Nadu, India: Initial perspectives based on E. coli release
Source: Int J Hyg Environ Health. 2022 Jun;243:113987. doi: 10.1016/j.ijheh.2022.113987 (PMC9227721; doi:10.1016/j.ijheh.2022.113987)
Supplement: Multimedia component 1 [file mmc1.docx]

## SUPPLEMENTARY INFORMATION

**Public Health Performance of Sanitation Technologies in Tamil Nadu, India: Initial Perspectives Based on *E. coli* Release**

Musa Manga ^1*^, Pete Kolsky^1^, Jan Willem Rosenboom^2^, Sudha Ramalingam^3^, Lavanya Sriramajayam^3^, Jamie Bartram^1,4^, Jill Stewart ^1^

^1^The Water Institute at UNC, Department of Environmental Sciences and Engineering, The Gillings School of Global Public Health, University of North Carolina at Chapel Hill, 4114 McGavran Hall, Campus Box # 7431, NC 27599, Chapel Hill, North Carolina, USA.

^2^Bill & Melinda Gates Foundation, Seattle, Washington, United States of America

^3^PSG Institute of Medical Sciences and Research, Off, Avinashi Rd, Masakalipalayam, Peelamedu, Coimbatore, Tamil Nadu 641004, India.

^4^School of Civil Engineering, University of Leeds, Leeds LS2 9JT, UK.

*^*^Corresponding email:* [*mmanga@email.unc.edu*](mailto:mmanga@email.unc.edu)

# Appendix A

# Appendix A-1: Methodology for Estimating Average Daily Flow Rate and Volume Discharge from Containment Systems

In cases where the containment system effluent pipe was easily accessible, the average daily effluent volume was measured using a graduated container. A 1000-gauge flexible plastic bag was used to collect the liquid discharge from tank effluent pipe for over a period of 24 hours (in 12 hourly intervals), and this was then transferred to the graduated container for measuring. The average daily discharge as effluent from the tank (in litres/day) was computed, based on the collected volume of liquid discharge (i.e. effluent) in a given time period. The flow measurements for each containment system were repeated weekly (the weekdays were representative of the entire week) for about 6 weeks.

In cases where the containment system effluent pipes were not easily accessible, the daily effluent discharge was estimated based on the hydrostatic or volume balance. Septic tanks operate in hydrostatic quasi-equilibrium where the volume of wastewater flowing into the tank is equal to the amount of treated wastewater pushed out of the tank through an outlet pipe [Missouri DHSS. (2018](#_ENREF_18)). We assumed in our study that all the containment systems discharging liquid effluent to the environment were in a hydraulic steady-state and that their inflow over a 24-hour period was equivalent to the outflow. Therefore, to estimate the average daily discharges, the containment systems with effluent pipes discharging liquid effluent to the environment, were partially emptied (i.e. about 1/3 of the volume of septage in the tank was removed). The level or depth of septage remaining within the containment system after partial emptying was then measured using a dipping stick, and the time recorded at which the measurement was taken (T = 0). After 24 hours, the level or depth of septage within the containment system was re-measured (T =1 day). This was repeated until the tank was full again. Considering the containment system dimensions (length, width, or diameter) measured previously, the volume of sludge or septage within the tank at T=0, T=1, and T=N were computed. Thereafter, the changes in the volume of septage within the tank (between T=0, T=1, and T=N) were computed, and these were then used to estimate the inflow or discharge rate of liquid effluent into the immediate environment. The minimum liquid detention time of each containment system was then computed as a quotient of the effective tank volume and the estimated flow rate (See Eq. 1). Further, the average daily discharge as the effluent release (q_D_) in litres/day was computed as a quotient of computed average volume discharge from the tank between T=0 and T=N, and the time in days (T=N).

Detention time (in days) = $\frac{Effective tank volume (litres)}{Estimated flow rate (litres/day)}$ ………………………………. Eq. 1

# Appendix B: Figures and Tables

Table S1 General usage and construction characteristics of on-site sanitation technologies observed in Trichy and NNP Coimbatore, Tamil Nadu, India

| **Characteristics/ description** | **Surveyed/ observed containment systems** | | | | | | | | | | | |
| --- | --- | --- | --- | --- | --- | --- | --- | --- | --- | --- | --- | --- |
|  | **Containment systems**  **(n = 178)** | | **Household Fully-lined Tanks**  **(n = 106)** | | | | **Community Toilet Fully-lined Tanks**  **(n = 28)** | | **Lined Tanks**  **(n = 25)** | | **Lined Pits**  **(n = 19)** | |
|  |  |  | **With Effluent Pipes (n=82)** | | **Without Effluent Pipes (n=24)** | |  |  |  |  |  |  |
|  | n | % | n | % | n | % | n | % | n | % | n | % |
| Primary means of greywater disposal for the household |  |  |  |  |  |  |  |  |  |  |  |  |
| Fully-lined / sealed tanks | 6 | 3.4% | 62 | 75.6% | 13 | 54.2% | 0 | 0.0% | 0 | 0.0% | 0 | 0.0% |
| Directly discharge to open drain | 126 | 70.8% | 13 | 15.9% | 9 | 37.5% | 25 | 89.3% | 18 | 72.0% | 8 | 42.1% |
| Directly to open ground | 42 | 23.6% | 5 | 6.1% | 1 | 4.2% | 3 | 10.7% | 7 | 28.0% | 10 | 52.6% |
| Soak pit | 4 | 2.2% | 2 | 2.4% | 1 | 4.2% | 0 | 0.0% | 0 | 0.0% | 1 | 5.3% |
|  |  |  |  |  |  |  |  |  |  |  |  |  |
| Sidewalls of the containment system fully plastered and impermeable |  |  |  |  |  |  |  |  |  |  |  |  |
| Yes | 159 | 89.3% | 82 | 100% | 24 | 100% | 28 | 100% | 25 | 100% | 0 | 0.0% |
| No | 19 | 10.7% | 0 | 0.0% | 0 | 0.0% | 0 | 0.0% | 0 | 0.0% | 19 | 100% |
| The bottom of the containment system fully sealed and impermeable |  |  |  |  |  |  |  |  |  |  |  |  |
| Yes | 134 | 75.3% | 82 | 100% | 24 | 100% | 28 | 100% |  | 0.0% |  | 0.0% |
| No material – open base/ bottom | 44 | 24.7% | 0 | 0.0% | 0 | 0.0% |  | 0.0% | 25 | 100% | 19 | 100% |
| The shape of the containment |  |  |  |  |  |  |  |  |  |  |  |  |
| Rectangular | 143 | 80.3% | 82 | 100% | 24 | 100% | 28 | 100% | 9 | 36.0% | 0 | 0.0% |
| Circular | 35 | 19.7% | 0 | 0.0% | 0 | 0.0% |  | 0.0% | 16 | 64.0% | 19 | 100% |
| Containment system constructed with effluent and/or overflow pipes |  |  |  |  |  |  |  |  |  |  |  |  |
| Yes | 109 | 61.2% | 82 | 100% | 24 | 100% | 27 | 96.4% | 0 | 0.0% | 0 | 0.0% |
| No | 69 | 38.8% | 0 | 0.0% | 0 | 0.0% | 1 | 3.6% | 25 | 100% | 19 | 100% |
| Effluent receivers of containment system discharge |  |  |  |  |  |  |  |  |  |  |  |  |
| Open drain | 79 | 72.5% | 67 | 81.7% | N/A | N/A | 12 | 44.4% | N/A | N/A | N/A | N/A |
| Stream, pond, or river | 5 | 4.6% | 2 | 2.4% | N/A | N/A | 3 | 11.1% | N/A | N/A | N/A | N/A |
| Soak away | 12 | 11.0% | 11 | 13.4% | N/A | N/A | 1 | 3.7% | N/A | N/A | N/A | N/A |
| Open ground | 13 | 11.9% | 2 | 2.4% | N/A | N/A | 11 | 40.7% | N/A | N/A | N/A | N/A |
|  |  |  |  |  |  |  |  |  |  |  |  |  |
| Length to Breadth Ratio |  |  |  |  |  |  |  |  |  |  |  |  |
| < 1.5 | 35 | 24.4% | 16 | 19.5% | 10 | 41.6% | 6 | 21.4% | 3 | 33.3% | N/A | N/A |
| 1.5 - 2.0 | 46 | 32.2% | 25 | 30.5% | 10 | 41.6% | 9 | 32.1% | 2 | 22.2% | N/A | N/A |
| > 2.0 | 62 | 43.4% | 41 | 50.0% | 4 | 16.6% | 13 | 46.4% | 4 | 44.4% | N/A | N/A |
| Sludge Depth |  |  |  |  |  |  |  |  |  |  |  |  |
| < 1.2 m | 36 | 20.2% | 5 | 6.1% | 2 | 8.3% | 1 | 3.6% | 14 | 56.0% | 14 | 73.7% |
| 1.2 - 1.5 m | 46 | 25.8% | 22 | 26.8% | 6 | 25.0% | 10 | 35.7% | 5 | 20.0% | 3 | 15.8% |
| 1.5 - 1.8 m | 38 | 21.3% | 19 | 23.2% | 7 | 29.2% | 6 | 21.4% | 5 | 20.0% | 1 | 5.3% |
| > 1.8 m | 58 | 32.6% | 36 | 43.9% | 9 | 37.5% | 11 | 39.3% | 1 | 4.0% | 1 | 5.3% |
| Effective tank volume per person |  |  |  |  |  |  |  |  |  |  |  |  |
| < 100 L | 22 | 12.4% | 0 | 0.0% | 0 | 0.0% | 16 | 57.2% | 3 | 12.0% | 3 | 15.8% |
| 100 - 250L | 38 | 21.3% | 7 | 8.5% | 1 | 4.2% | 9 | 32.1% | 10 | 40.0% | 11 | 57.9% |
| 250 - 500L | 26 | 14.6% | 13 | 15.8% | 4 | 16.7% | 2 | 7.1% | 3 | 12.0% | 4 | 21.1% |
| > 500 L | 92 | 51.6% | 62 | 75.6% | 19 | 79.2% | 1 | 3.6% | 9 | 26.0% | 1 | 5.3% |

Table S2: Physical measurements of the containment systems

| **Containment system physical measurements, characteristics and usage** | **Surveyed/ observed containment systems** | | | | | | | | | **p-value for differences between lined tanks** |
| --- | --- | --- | --- | --- | --- | --- | --- | --- | --- | --- |
|  | **Household Fully-lined Tanks (n = 106)** | | **Community Toilets**  **(n = 28)** | | **Lined Tanks**  **(n=25)** | | | **Lined Pits**  **(n=19)** | |  |
|  | **Range | Mean ± SD | **Range | Mean ± SD | **Range | Mean ± SD | **Range | | Mean ± SD |  |
| Lengths (m) | 1.2 - 5.5 | 2.7±0.8 | 1.7 - 7.3 | 4.3±1.5 | 2.0 - 3.2  (n=9) | 2.5 ± 0.4 | N/A | | N/A | 0.00001 |
| Width (m) | 0.7 - 2.7 | 1.4±0.4 | 1.2 - 3.1 | 2.1±0.6 | 0.8-1.7  (n=9) | 1.4 ± 0.3 | N/A | | N/A | 0.00001 |
| Diameter (m) | N/A | N/A | N/A | N/A | 0.6-1.0  (n=16) | 0.9 ± 0.12 | 0.9 - 1.5 | | 1.0±0.1 | 0.033 |
| Length to Breath (L:B) Ratio | 1.09-5 | 2.0±0.7 | 1.1-4.0 | 2.1±0.7 | 1.3-3.2  (n=9) | 1.9±0.6 | N/A | | N/A | 0.6912 |
| Effective depth (m) | 1.0-3.3 | 1.8±0.5 | 1.1-2.6 | 1.7+0.4 | 0.8-3.0 | 1.5±0.5 (n=25) | 0.3-2.9 | | 1.3±0.7 (n=19) | 0.0003 |
| Effective tank volume (m^3^) | 1.8-21.9 | 6.9±4.2 | 3.7-38.2 | 16.8±8.5 | 0.2-14.5 | 1.0* | 0.2-5.1 | | 0.8* | 0.00001 |

*Median, **Range= Minimum value – Maximum value, and SD=Standard deviation.

Table S3: Key design and operational characteristics of on-site sanitation technologies observed in Trichy and NNP Coimbatore, Tamil Nadu, India

| **Characteristics/ description** | **Surveyed/ observed containment systems** | | | | | | | | | | | |
| --- | --- | --- | --- | --- | --- | --- | --- | --- | --- | --- | --- | --- |
|  | **Containment systems** | | **Household Fully-lined Tanks**  **(n = 106)** | | | | **Community Toilet Fully-lined Tanks**  **(n = 28)** | | **Lined Tanks**  **(n = 25)** | | **Lined Pits**  **(n = 19)** | |
|  |  |  | **With Effluent Pipes (n=82)** | | **Without Effluent Pipes (n=24)** | |  |  |  |  |  |  |
|  | n | % | n | % |  |  | n | % | n | % | n | % |
| Hydraulic/ liquid Retention time |  |  |  |  |  |  |  |  |  |  |  |  |
| <6 hours | 25 | 32.5% | 9 | 17.0% | N/A | N/A | 16 | 66.7% | N/A | N/A | N/A | N/A |
| 6 - 24 hours | 43 | 55.8% | 35 | 66.1% | N/A | N/A | 8 | 33.4% | N/A | N/A | N/A | N/A |
| > 24 hours | 9 | 11.7% | 9 | 17.0% | N/A | N/A | 0 | 0.0% | N/A | N/A | N/A | N/A |
| The containment system has ever filled-up |  |  |  |  |  |  |  |  |  |  |  |  |
| Yes | 146 | 82.0% | 76 | 92.7% | 22 | 91.7% | 28 | 100% | 9 | 36.0% | 11 | 57.9% |
| No | 30 | 16.9% | 6 | 7.3% | 2 | 8.3% | 0 | 0.0% | 14 | 56.0% | 8 | 42.1% |
| Don't Know | 2 | 1.1% | 0 | 0 | 0 | 0 | 0 | 0.0% | 2 | 8.0% | 0 | 0.0% |
| Containment system has ever been emptied in 5 years |  |  |  |  |  |  |  |  |  |  |  |  |
| Yes | 146 | 82.0% | 75 | 91.5% | 22 | 91.7% | 28 | 100% | 10 | 40.0% | 11 | 57.9% |
| No | 32 | 18.0% | 7 | 8.5% | 2 | 8.3% | 0 | 0.0% | 15 | 60.0% | 8 | 42.1% |
| Emptying Frequency of the containment systems in 5 years |  |  |  |  |  |  |  |  |  |  |  |  |
| <2 | 30 | 20.5% | 18 | 24.0% | 0 | 0 | 2 | 7.1% | 3 | 30.0% | 7 | 63.6% |
| 2 - 10 | 70 | 47.9% | 49 | 65.3% | 12 | 54.5% | 1 | 3.6% | 4 | 40.0% | 4 | 36.4% |
| 10 - 30 | 25 | 17.1% | 8 | 10.7% | 8 | 36.4% | 7 | 25.0% | 2 | 20.0% | 0 | 0.0% |
| > 30 | 21 | 14.4% | 0 | 0.0% | 2 | 9.1% | 18 | 64.2% | 1 | 10.0% | 0 | 0.0% |
| Storage periods/ Detention period of human waste before emptying |  |  |  |  |  |  |  |  |  |  |  |  |
| < 4 weeks | 16 | 11.0% | 0 | 0 | 1 | 4.5% | 15 | 53.6% | 0 | 0.0% | 0 | 0.0% |
| 1 – 6 months | 17 | 11.6% | 5 | 6.7% | 4 | 18.2% | 8 | 28.6% | 0 | 0.0% | 0 | 0.0% |
| 0.5 - 1.5 years | 55 | 37.7% | 29 | 38.7% | 12 | 54.5% | 4 | 14.3% | 8 | 80.0% | 2 | 18.2% |
| 1.5 - 3 years | 37 | 25.3% | 26 | 34.7% | 5 | 22.7% | 1 | 3.6% | 2 | 20.0% | 3 | 27.3% |
| > 3.0 years | 21 | 14.4% | 15 | 20.0% | 0 | 0.0% | 0 | 0.0% | 0 | 0.0% | 6 | 54.5% |

Table S4: Pairwise Post-hoc Tukey test results for effective tank volume per capita, storage periods and emptying frequency of the observed containment systems

| **Sanitation technologies compared** | **Effective tank volume per capita** | | | **Storage periods between emptying** | | | **Emptying Frequency** | | | |
| --- | --- | --- | --- | --- | --- | --- | --- | --- | --- | --- |
|  | **Coefficient** | **95% CI** | **p-value** | **Coefficient** | **95% CI** | **p-value** | **Coefficient** | **95% CI** | **p-value** |  |
| Household Fully-lined tanks without effluent pipes **vs** Household Fully-lined tanks with effluent pipes | -0.1 | -0.5, 0.4 | 0.99 | -12.9 | -46.7, 21.0 | 0.92 | 11.6 | -113.4, 136.5 | 0.99 |  |
| Community Fully-lined tanks **vs** Household Fully-lined tanks with effluent pipes | -1.1 | -1.5, -0.7 | 0.0001* | -11.2 | -41.0, 18.6 | 0.92 | 210.0 | 101.7, 318.2 | 0.0001* |  |
| Lined tanks **vs** Household Fully-lined tanks with effluent pipes | -0.7 | -1.2, -0.3 | 0.0001* | 48.7 | 17.6, 79.8 | 0.0001* | -3.4 | -159.4, 166.4 | 0.991 |  |
| Lined pits **vs** Household Fully-lined tanks with effluent pipes | -1.0 | -1.5, -0.5 | 0.0001* | 45.0 | 10.5, 79.6 | 0.003* | -2.0 | -158.5, 154.3 | 0.991 |  |
| Community Fully-lined tanks **vs** Household Fully-lined tanks without effluent pipes | -1.0 | -1.6, -0.5 | 0.0001* | 1.7 | -37.5, 40.8 | 0.995 | 198.4 | 55.7, 341.1 | 0.002* |  |
| Lined tanks **vs** Household Fully-lined tanks without effluent pipes | -0.7 | -1.2, -0.8 | 0.017* | 61.6 | 21.4, 101.7 | 0.0001* | -8.1 | -195.6, 179.5 | 0.99 |  |
| Lined pits **vs** Household Fully-lined tanks without effluent pipes | -0.9 | -1.5, -0.3 | 0.0001* | 57.4 | 15.0, 100.8 | 0.002* | -13.5 | -195.4, 168.3 | 0.99 |  |
| Lined tanks **vs** Community Fully-lined tanks | -0.4 | -0.2, 0.9 | 0.305 | 59.9 | 23.1, 96.7 | 0.0001* | -206.5 | -383.3, -29.7 | 0.013* |  |
| Lined pits **vs** Community Fully-lined tanks | 0.9 | -0.5, 0.7 | 0.99 | 56.2 | 16.5, 96.0 | 0.001* | -212.0 | -382.8, -41.2 | 0.007* |  |
| Lined pits **vs** Lined tanks | -0.3 | -0.9, 0.3 | 0.68 | -3.7 | -44.3, 37.1 | 0.995 | -5.5 | -215.2, 204.3 | 0.99 |  |

*Significantly different at 95% Confidence Level

Table S5: Pairwise Post-hoc Tukey test results for average daily per capita *E.coli* release from the different sanitation technologies

| **Sanitation technologies compared** | **Average daily *E. coli* release per capita by discharges (i.e. overflows and/or effluents)** | | | **Average daily *E. coli* release per capita due to periodic desludging** | | | | **Average daily combined per capita *E. coli* release** | | | |
| --- | --- | --- | --- | --- | --- | --- | --- | --- | --- | --- | --- |
|  | **Coefficient** | **95% CI** | **p-value** | **Coefficient** | **95% CI** | **p-value** | **Coefficient** | | **95% CI** | **p-value** |  |
| Household Fully-lined tanks with effluent pipes to Soakaway **vs** Household Fully-lined tanks with effluent pipes to Environment | N/A | N/A | N/A | 0.02 | -1.4, 1.4 | 0.995 | -1.7 | | -3.0, -0.5 | 0.001* |  |
| Household Fully-lined tanks without effluent pipes **vs** Household Fully-lined tanks with effluent pipes to Environment | N/A | N/A | N/A | 1.9 | 0.8, 3.1 | 0.0001* | 0.2 | | -0.8, 1.2 | 0.99 |  |
| Community Fully-lined tanks **vs** Household Fully-lined tanks with effluent pipes to Environment | 0.4 | -0.2, 1.0 | 0.363 | 1.7 | 0.8, 2.6 | 0.0001* | 0.8 | | 0.01, 1.5 | 0.045* |  |
| Lined tanks **vs** Household Fully-lined tanks with effluent pipes to Environment | N/A | N/A | N/A | -0.3 | -1.8, 1.2 | 0.994 | -2.0 | | -3.4, -0.7 | 0.0001* |  |
| Lined pits **vs** Household Fully-lined tanks with effluent pipes to Environment | N/A | N/A | N/A | -1.6 | -3.0, -0.2 | 0.016* | -3.4 | | -4.6, -2.1 | 0.0001* |  |
| Straight pipes **vs** Household Fully-lined tanks with effluent pipes to Environment | 1.8 | 1.2, 2.4 | 0.0001* | N/A | N/A | N/A | 1.8 | | 1.0, 2.5 | 0.0001* |  |
| Sanitary sewer **vs** Household Fully-lined tanks with effluent pipes to Environment | 1.5 | 0.9, 2.2 | 0.0001* | N/A | N/A | N/A | 1.5 | | 0.8, 2.3 | 0.0001* |  |
|  |  |  |  |  |  |  |  | |  |  |  |
| Household Fully-lined tanks without effluent pipes **vs** Household Fully-lined tanks with effluent pipes to soakaway | N/A | N/A | N/A | 1.9 | 0.2, 3.6 | 0.015* | 1.9 | | 0.5, 3.4 | 0.002* |  |
| Community Fully-lined tanks **vs** Household Fully-lined tanks with effluent pipes to soakaway | N/A | N/A | N/A | 1.7 | 0.2, 3.2 | 0.018* | 2.5 | | 1.2, 3.8 | 0.0001* |  |
| Lined tanks **vs** Household Fully-lined tanks with effluent pipes to soakaway | N/A | N/A | N/A | -0.3 | -2.3, 1.6 | 0.997 | -0.3 | | -2.0, 1.4 | 0.99 |  |
| Lined pits **vs** Household Fully-lined tanks with effluent pipes to soakaway | N/A | N/A | N/A | -1.6 | -3.5, 0.2 | 0.124 | -1.6 | | -3.3, -0.01 | 0.047* |  |
| Straight pipes **vs** Household Fully-lined tanks with effluent pipes to soakaway | N/A | N/A | N/A | N/A | N/A | N/A | 3.5 | | 2.2, 4.8 | 0.0001* |  |
| Sanitary sewer **vs** Household Fully-lined tanks with effluent pipes to soakaway | N/A | N/A | N/A | N/A | N/A | N/A | 3.3 | | 1.9, 4.6 | 0.0001* |  |
|  |  |  |  |  |  |  |  | |  |  |  |

Table S5 (Cont’d): Pairwise Post-hoc Tukey test results for average daily per capita *E.coli* release from the different sanitation technologies

| **Sanitation technologies compared** | **Average daily *E. coli* release per capita by discharges (i.e. overflows and/or effluents)** | | | **Average daily *E. coli* release per capita due to periodic desludging** | | | | **Average daily combined per capita *E. coli* release** | | | |
| --- | --- | --- | --- | --- | --- | --- | --- | --- | --- | --- | --- |
|  | **Coefficient** | **95% CI** | **p-value** | **Coefficient** | **95% CI** | **p-value** | **Coefficient** | | **95% CI** | **p-value** |  |
| Community Fully-lined tanks **vs** Household Fully-lined tanks without effluent pipes | N/A | N/A | N/A | -0.2 | -1.5, 1.0 | 0.996 | 0.6 | | -0.5, 1.7 | 0.704 |  |
| Lined tanks **vs** Household Fully-lined tanks without effluent pipes | N/A | N/A | N/A | -2.2 | -4.0, -0.5 | 0.005* | -2.2 | | -3.7, -0.7 | 0.0001* |  |
| Lined pits **vs** Household Fully-lined tanks without effluent pipes | N/A | N/A | N/A | -3.6 | -5.2, 1.9 | 0.0001* | -3.6 | | -5.0, -2.1 | 0.0001* |  |
| Straight pipes **vs** Household Fully-lined tanks without effluent pipes | N/A | N/A | N/A | N/A | N/A | N/A | 1.6 | | 0.5, 2.6 | 0.0001* |  |
| Sanitary sewer **vs** Household Fully-lined tanks without effluent pipes | N/A | N/A | N/A | N/A | N/A | N/A | 1.4 | | 0.3, 2.4 | 0.005* |  |
|  |  |  |  |  |  |  |  | |  |  |  |
| Lined tanks **vs** Community Fully-lined tanks | N/A | N/A | N/A | -2.0 | -3.6, -0.4 | 0.006* | -2.8 | | -4.2, -1.4 | 0.0001* |  |
| Lined pits **vs** Community Fully-lined tanks | N/A | N/A | N/A | -3.3 | -4.8, -1.8 | 0.0001* | -4.1 | | -5.4, -2.8 | 0.0001* |  |
| Straight pipes **vs** Community Fully-lined tanks | 1.4 | 0.7, 2.1 | 0.0001* | N/A | N/A | N/A | 1.0 | | 0.1, 1.9 | 0.016* |  |
| Sanitary sewer **vs** Community Fully-lined tanks | 1.2 | 0.4, 1.9 | 0.0001* | N/A | N/A | N/A | 0.8 | | -0.1, 1.7 | 0.148 |  |
|  |  |  |  |  |  |  |  | |  |  |  |
| Lined pits **vs** Lined tanks | N/A | N/A | N/A | -1.3 | -3.3, 0.6 | 0.365 | -1.3 | | -3.0, 0.4 | 0.244 |  |
| Straight pipes **vs** Lined tanks | N/A | N/A | N/A | N/A | N/A | N/A | 3.8 | | 2.4, 5.2 | 0.0001* |  |
| Sanitary sewer **vs** Lined tanks | N/A | N/A | N/A | N/A | N/A | N/A | 3.6 | | 2.2, 5.0 | 0.0001* |  |
|  |  |  |  |  |  |  |  | |  |  |  |
| Straight pipes **vs** Lined pits | N/A | N/A | N/A | N/A | N/A | N/A | 5.1 | | 3.8, 6.4 | 0.0001* |  |
| Sanitary sewer **vs** Lined pits | N/A | N/A | N/A | N/A | N/A | N/A | 4.9 | | 3.6, 6.2 | 0.0001* |  |
| Sanitary sewer **vs** Straight pipes | -0.2 | -0.9, 0.5 | 0.858 | N/A | N/A | N/A | -0.2 | | -1.1, 0.7 | 0.995 |  |

N/A – Not Applicable

Table S6: Regression results of design, construction and operational features of different containment system parameters with average daily per capita *E. coli* release

| **Design, construction and operation features** | **Average daily *E. coli* release per capita due to overflows and/or effluents** | | | | |  | | **Average daily *E. coli* release per capita due to periodic desludging** | | | |  | **Average daily combined per capita *E. coli* release** | | | |
| --- | --- | --- | --- | --- | --- | --- | --- | --- | --- | --- | --- | --- | --- | --- | --- | --- |
|  | **No. of Observ.** | **Coefficient** | **R^2^** | ***p*-value** |  | | **No. of Observ.** | | **Coefficient** | **R^2^** | ***p*-value** |  | **No. of Observ.** | **Coefficient** | **R^2^** | ***p*-value** |
| Liquid Retention time (days)* | 125 | -1.43 | 0.28 | 0.00001* |  | | 70 | | -0.8750 | 0.08 | 0.0207* |  | 118 | -1.45 | 0.27 | 0.00001* |
| Effective tank volume per capita | 77 | N/A | 0.01 | 0.8400 |  | | 109 | | N/A | 0.01 | 0.861 |  | 104 | N/A | 0.03 | 0.0687* |
| Storage periods of human waste before emptying (years) | 124 | -0.48 | 0.23 | 0.00001* |  | | 103 | | -0.68 | 0.30 | 0.00001* |  | 150 | -0.72 | 0.30 | 0.00001* |
| Emptying frequency in the past 5 years | 70 | N/A | 0.03 | 0.186 |  | | 103 | | 0.04 | 0.22 | 0.00001* |  | 102 | 0.03 | 0.12 | 0.0003* |
| Number of users | 102 | N/A | 0.01 | 0.8416 |  | | 103 | | 0.005 | 0.13 | 0.0001* |  | 127 | 0.003 | 0.02 | 0.047* |

N/A – Not Applicable; *Significantly different at 95% Confidence Level.

Table S7: Regression results of design, construction and operational features of fully-lined tanks with average daily per capita *E. coli* release

| **Design, construction and operation features** | **Average daily *E. coli* release per capita due to overflows and/or effluents** | | | |  | **Average daily *E. coli* release per capita due to periodic desludging** | | | |  | **Average daily combined per capita *E. coli* release** | | | | |
| --- | --- | --- | --- | --- | --- | --- | --- | --- | --- | --- | --- | --- | --- | --- | --- |
| **Design Features** | **No. of Observ.** | **Coefficient** | **R^2^** | ***p*-value** |  | **No. of Observ.** | **Coefficient** | **R^2^** | ***p*-value** |  | | **No. of Observ.** | **Coefficient** | **R^2^** | ***p*-value** |
| Effective tank volume per capita | 77 | N/A | 0.01 | 0.8400 |  | 90 | N/A | 0.01 | 0.491 |  | | 89 | -0.36 | 0.06 | 0.018* |
| Liquid Retention time (days) | 77 | -0.71 | 0.11 | 0.0033* |  | 70 | -0.88 | 0.07 | 0.0207* |  | | 70 | -0.83 | 0.13 | 0.0025* |
| Inlet and outlet pipe configuration | 76 | **-**1.09 | 0.28 | 0.00001* |  | N/A | N/A | N/A | N/A |  | | 80 | -0.89 | 0.25 | 0.0001* |
| Number of Chamber | 77 | N/A | 0.01 | 0.6279 |  | 90 | N/A | 0.01 | 0.5203 |  | | 89 | N/A | 0.01 | 0.3470 |
| Length to Breadth Ratio | 77 | N/A | 0.01 | 0.4626 |  | 90 | N/A | 0.01 | 0.8003 |  | | 89 | N/A | 0.01 | 0.7840 |
| Septage depth | 77 | N/A | 0.01 | 0.4574 |  | 90 | N/A | 0.01 | 0.8858 |  | | 89 | N/A | 0.01 | 0.5840 |
| Disposal of the liquid effluent Soakaways | N/A | N/A | N/A | N/A |  | 77 | N/A | 0.01 | 0.960 |  | | 76 | -1.48 | 0.14 | 0.001* |
|  |  |  |  |  |  |  |  |  |  |  | |  |  |  |  |
| **Operational Characteristics** |  |  |  |  |  |  |  |  |  |  | |  |  |  |  |
| Storage periods of human waste before emptying (years) | N/A | N/A | N/A | N/A |  | 90 | -0.71 | 0.35 | 0.00001* |  | | 89 | -0.48 | 0.21 | 0.00001* |
| Emptying frequency in the past 5 years | N/A | N/A | N/A | N/A |  | 90 | 0.03 | 0.26 | 0.00001* |  | | 89 | 0.03 | 0.15 | 0.0001* |
| Number of users | 77 | 0.002 | 0.04 | 0.089 |  | 90 | 0.004 | 0.11 | 0.0016* |  | | 89 | 0.003 | 0.09 | 0.0042* |

N/A – Not Applicable; *Significantly different at 95% Confidence Interval

Table S8: Raw, and intermediate variables for computation of average daily per capita *E. coli* release from different sanitation Technologies

| Sanitation Technologies | E. *coli c*oncentrations in overflow and/or effluent – (Log_10_ E. *coli* per L)  Arithmetic Mean (95% CI) | Average daily Per capita liquid overflows and/or effluent– (Litres per capita per day)  Arithmetic means (95% CI) | Average daily *E. coli* release due to overflows and/or effluent (Log_10_ *E. coli* per day)  Arithmetic Mean (95% CI) | Average daily *E. coli* release due to overflows and/or effluent (Log_10_ *E. coli* per capita per day)  Arithmetic means (95% CI) |  | E. *coli c*oncentration of sludge/ Septage in tank (Log_10_ *E. coli*)  Arithmetic means (95% CI) | Volume of sludge/ septage in the Containment system prior to emptying (M^3^)  Arithmetic means (95% CI) | *E. coli* load in septage in containment system prior to emptying (Log_10_ E. *coli*)  Arithmetic means (95% CI) | Average daily *E. coli* release due to periodic desludging (Log_10_ *E. coli* per day)  Arithmetic means (95%CI) | Average daily *E. coli* release due to periodic desludging (Log_10_ *E. coli* per capita per day)  Arithmetic means (95%CI) |  | Average combined daily *E. coli* release (Log_10_ *E. coli* per day)  Arithmetic means (95%CI) | Average daily Combined Per capita *E. coli* release (Log_10_ *E. coli* per capita per day)  Arithmetic means (95%CI) |
| --- | --- | --- | --- | --- | --- | --- | --- | --- | --- | --- | --- | --- | --- |
| Household Fully-lined Tanks without effluent pipes | N/A | N/A | N/A | N/A |  | 7.7 (7.2, 8.3) | 6.8 (5.2, 8.4) | 11.7 (11.0, 12.3) | 9.8 (9.3, 10.4) | 9.0 (8.4, 9.5) |  | 9.8 (9.3, 10.4) | 9.0 (8.4, 9.5) |
| Household Fully-lined Tanks with overflow/ effluent pipes to soakaway | N/A | N/A | N/A | N/A |  | 6.7 (5.4, 7.9) | 10.6 (6.5, 14.7) | 10.6 (9.5, 11.7) | 7.7 (6.5, 8.9) | 7.0 (5.8, 8.3) |  | 7.7 (6.5, 8.9) | 7.0 (5.8, 8.3) |
| Household Fully-lined Tanks with overflow/ effluent pipes to environment | 7.0 (6.7, 7.4) | 69 (57.2, 80.6) | 9.6 (9.3, 9.9) | 8.8 (8.5, 9.0) |  | 6.8 (6.5, 7.2) | 5.2 (4.6, 6.0) | 10.7 (10.4, 10.9) | 7.8 (7.5, 8.2) | 7.0 (6.7, 7.3) |  | 9.6 (9.3, 9.9) | 8.8 (8.5, 9.1) |
| Community Toilet Fully-lined Tank | 7.9 (7.5, 8.2) | 20.2 (16.7, 23.6) | 11.4 (11.0, 11.8) | 9.1 (8.8, 9.5) |  | 8.2 (7.8, 8.5) | 16.5 (12, 20.3) | 12.3 (11.9, 12.7) | 11.0 (10.4, 11.6) | 8.7 (8.1, 9.3) |  | 11.8 (11.3, 12.2) | 9.2 (9.1, 10.0) |
| Lined Tank | N/A | N/A | N/A | N/A |  | 6.7 (6.3,7.0) | 1.7 (0.7, 2.8) | 9.6 (9.3, 10.0) | 7.4 (6.7, 8.1) | 6.7 (6.1, 7.3) |  | 7.4 (6.7, 8.1) | 6.7 (6.1, 7.3) |
| Lined Pit | N/A | N/A | N/A | N/A |  | 5.5 (5.0,6.0) | 0.8 (0.5, 1.1) | 8.4 (7.8, 8.9) | 6.2 (5.4, 6.9) | 5.4 (4.7, 6.1) |  | 6.2 (5.4, 6.9) | 5.4 (4.7, 6.1) |
| Black-pipes | 9.0 (8.7, 9.3) | 42 (30.1, 53.1) | 11.3 (10.9, 11.6) | 10.5 (10.2, 10.8) |  | N/A | N/A | N/A | N/A | N/A |  | 11.3 (10.9, 11.6) | 10.5 (10.2, 10.8) |
| Sanitary Sewer | 8.8 (8.4,9.2) | 32.4 | 11.0 (10.6, 11.4) | 10.3 (9.9, 10.7) |  | N/A | N/A | N/A | N/A | N/A |  | 11.0 (10.6, 11.4) | 10.3 (9.9, 10.7) |

Table S9: Multiple regression analysis results of key design feature and operation practices of the septic tanks/ FTand average combined daily *E. coli* release

| **Design features and** | **Coef. (**Mean Log_10_ Reduction**)** | ***P*** |
| --- | --- | --- |
| Inlet and outlet pipes configuration | -0.892 | 0.0001 |
| Liquid retention time (days) | -0.563 | 0.011 |
| Storage periods of human waste (years) | -0.180 | 0.026 |
| Soakaway | -0.903 | 0.029 |


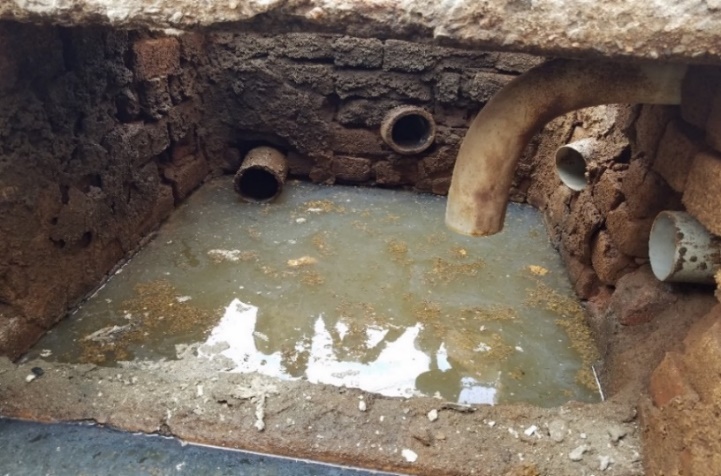

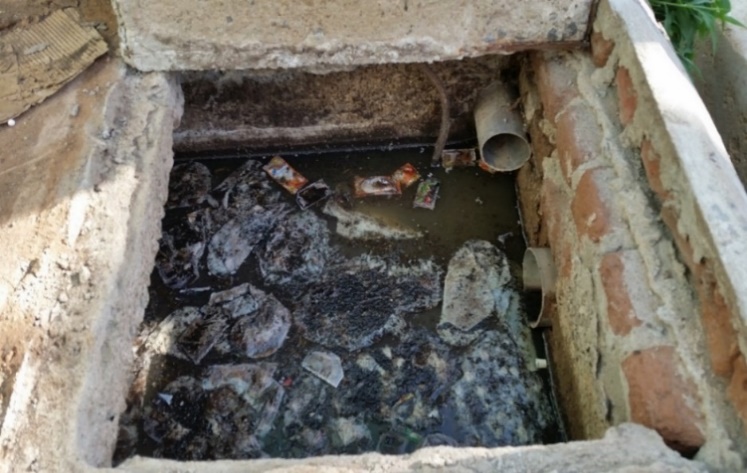


**Figure S1:** Fully-lined tanks encountered in the study communities constructed with wrong inlet and effluent pipe configuration; with the inlet pipe adjacent to outlet pipe.

# Appendix C: List of Survey Questions

Table S10: User (Household) Survey Questions

| **Qn. No.** | **Survey Question** |  | **Survey Responses** |
| --- | --- | --- | --- |
| **Part 1: - Use of Sanitation Infrastructure** | |  |  |
|  | What toilet facility does your household member use? |  | - On plot – Household private toilet |
|  |  |  | - On plot – Shared toilet |
|  |  |  | - Off plot – Community toilet |
|  |  |  | - Off plot – Public toilet |
|  |  |  | - Off plot – Establishment (e.g. Hotels, Institutions) |
|  | What kind of toilet facility do members of your household usually use? |  | - Automatic Flush |
|  |  |  | - Cistern flush |
|  |  |  | - Pour/manual flush |
|  |  |  | - Ventilated improved pit latrine |
|  |  |  | - Pit latrine with slab |
|  |  |  | - Others (specify):______________________ |
|  |  |  | - No facilities or bush or field |
|  | Where do the contents of toilet discharge to? |  | - Sanitary Sewer |
|  |  |  | - Septic tank |
|  |  |  | - Fully-lined tanks with effluent pipe |
|  |  |  | - Fully-lined tanks without effluent pipe |
|  |  |  | - Holding tanks/ Cesspits |
|  |  |  | - Lined tank with impermeable side walls and permeable base |
|  |  |  | - Lined pit with permeable side walls and base |
|  |  |  | - Direct discharge to the environment |
|  |  |  | - Others (specify): _____________________ |
|  |  |  | - Don’t Know |
|  | Does the containment system that receive the toilet content has an effluent pipe? |  | - Yes |
|  |  |  | - No |
|  |  |  | - Don’t Know |
|  | Where does the effluent pipe from the containment system discharge to? |  | - Soakaways |
|  |  |  | - Drain field |
|  |  |  | - Environment (Open drain, open ground, surface water) |
|  |  |  | - Others(specify):________________________ |
|  |  |  | - Don’t Know |

Table S10 (Cont’d): User (Household) Survey Questions

| **Qn. No.** | **Survey Question** |  | **Survey Responses** |
| --- | --- | --- | --- |
|  | At home, where do you dispose of greywater (wastewater from kitchen, bathroom, and laundry)? |  | - Piped sewer system |
|  |  |  | - On-site sanitation system |
|  |  |  | - Soakpit or drain field |
|  |  |  | - Directly to open drain / ditch |
|  |  |  | - Directly to lake or river |
|  |  |  | - Directly to open ground |
|  |  |  | - Others (specify): |
|  |  |  | - Don’t Know |
| **Part 2: - Use of the Toilet Facility** | | | |
|  | Do you share your toilet facility with other households? |  | - Yes |
|  |  |  | - No |
|  |  |  | - Open Defecation |
|  | How many other households share this toilet facility? |  | - [enter number] |
|  |  |  | - Don’t Know |
|  | Do **ALL** household members use the toilet exclusively when they are at home? |  | - Yes |
|  |  |  | - No, we also practice open defecation |
|  |  |  | - No, we also use community toilet |
|  |  |  | - Others (Specify): _________________________ |
|  | How many **people** use this toilet facility regularly? |  | - [enter number] |
|  |  |  | - Don’t Know |
|  | Can any member of the public use this toilet facility? |  | - Yes |
|  |  |  | - No |
|  | Where is this toilet facility located? |  | - Inside the household or compound |
|  |  |  | - Outside the household or compound |
|  |  |  | - Others (Please Specify) ___________________ |
|  | **Enumerator -** place the toilet in one of these categories based on the answers |  | - On plot – Household private |
|  |  |  | - On plot – Shared |
|  |  |  | - Off plot – Communal |
|  |  |  | - Off plot – Public |
|  |  |  | - Off plot – Establishment (e.g. Hotels, Institutions) |

Table S10 (Cont’d): Users (Household) Survey Questions

| **Qn. No.** | **Survey Question** |  | **Survey Responses** |
| --- | --- | --- | --- |
| **Part 3: - Filling up and Desludging of the Containment System** | | | |
|  | In which year was this toilet facility or containment system built? |  | - [Enter Number of Years] |
|  |  |  | - Don’t Know |
|  | How many years ago was this toilet facility or containment system built? |  | - [Enter Number of Years] |
|  |  |  | - Don’t Know |
|  | Has this containment system ever filled up or required emptying? |  | - Yes |
|  |  |  | - No |
|  |  |  | - Don’t Know |
|  | What did you do when the containment system (pit/tank) filled-up last time? |  | - Attempted to clear a blockage |
|  |  |  | - Emptied and reused pit/tank |
|  |  |  | - Abandoned and pit/tank unsealed |
|  |  |  | - Broke the tank/pit to release contents to surface or drain |
|  |  |  | - Made structural improvements to the toilet or tank/pit |
|  |  |  | - Abandoned with sealed cover on pit/tank |
|  |  |  | - Covered and used alternative pit |
|  |  |  | - Others (Specify) |
|  |  |  | - Don’t Know |
|  | Has the containment system (pit/tank) ever been emptied in the last 5 years? |  | - Yes |
|  |  |  | - No |
|  | How many times has the containment system (pit/tank) been emptied in the last 5 years? |  | - [enter number] |
|  |  |  | - Don’t Know |
|  | When was the containment system (pit/tank) last emptied (month and year)? Write as MM [Month] & YYYY [Year] |  | - …….Month …….year |
|  |  |  | - Don’t Know |
|  | On average, how long does it take for the emptied containment system (pit/tank) to fill up or require desludging again? |  | - [Enter number] Enter Days, Weeks, Months or Years |
|  |  |  | - Don’t Know |

Table S11: Observation Survey Checklist

| **PART 1: - Unsafe Releases Associated with Operation of the Containment system** | | | |
| --- | --- | --- | --- |
| **Qn. No** | **Questions** |  | **Survey Observations** |
|  | Is the containment system (pit/ tank) or effluent receiver (e.g. soakaway, open drain, etc.) covered and the cover slab sealed well? |  | - Not covered |
|  |  |  | - Covered but not sealed well |
|  |  |  | - Covered and sealed well |
|  |  |  | - Other (specify): __________________ |
|  |  |  | - Don’t Know |
|  | Is the containment system (pit/ tank) or effluent receiver (e.g soakaway, open drain, etc.) full, overflowing or allowing exfiltrated liquid or faecal waste to leak onto the surface? |  | - Overflowing or leaking |
|  |  |  | - Full, but not overflowing or leaking |
|  |  |  | - Not full or leaking |
|  |  |  | - Other (specify): __________________ |
|  |  |  | - Don’t Know |
|  | Is there evidence that the containment system (pit/tank) needs desludging? |  | - Pit/ Tank full/ overflowing and in need of desludging immediately |
|  |  |  | - Pit/ Tank not full/overflowing but likely to need desludging soon |
|  |  |  | - Pit/ Tank not full/overflowing and unlikely to need desludging soon |
|  |  |  | - Other (specify): ___________________________ |
|  |  |  | - Don’t Know |
|  | Is there evidence that the containment system has overflowed before? |  | - Strong evidence of overflow, with excreta still visible |
|  |  |  | - Some evidence of overflow, but excreta not that visible |
|  |  |  | - No evidence of overflow |
|  |  |  | - Other (specify):   _______________________________ |
|  |  |  | - Don’t Know |
|  | Is the household toilet or containment system connected to the open drain? |  | - Yes |
|  |  |  | - No |

Table S11(Cont’d): Observation Survey Checklist

| **Qn. No** | **Question** |  | | **Response** | | |  |
| --- | --- | --- | --- | --- | --- | --- | --- |
| **PART 2: - Characterization of the Desludged Containment system** | | | | | | |  |
|  | What is the type of the containment system being emptied? |  | | | | - Septic tank | |
|  |  |  | | | | - Fully-lined tanks with effluent pipe | |
|  |  |  | | | | - Fully-lined tanks without effluent pipe | |
|  |  |  | | | | - Holding tanks/ Cesspits | |
|  |  |  | | | | - Lined pit with impermeable side walls and permeable base | |
|  |  |  | | | | - Lined pit with permeable walls and open bottom | |
|  |  |  | | | | - Others (specify): ___________________________ | |
|  |  |  | | | | - Don’t Know | |
|  | What were the material(s) used for construction of side walls of the fully-emptied containment system? **[Multiple response]** | |  | | - Stone or Rubble | |  |
|  |  |  |  | | - Burnt Brick | |  |
|  |  |  |  | | - Plain Cement Concrete (PCC) or Reinforced Cement Concrete (RCC) | |  |
|  |  |  |  | | - Fiber Reinforced Plastic or hard plastic | |  |
|  |  |  |  | | - Pre-cast RCC Slabs | |  |
|  |  |  |  | | - Pre-cast RCC Rings | |  |
|  |  |  |  | | - Stone Slabs | |  |
|  |  |  |  | | - Honeycombed bricks | |  |
|  |  |  |  | | - No material | |  |
|  |  |  |  | | - Others (Specify): ________________ | |  |
|  | Are the side walls of the containment system fully plastered and impermeable? | |  | | - Yes | |  |
|  |  |  |  | | - No | |  |
|  |  |  |  | | - Others (Specify): __________________ | |  |
|  | What is the construction material used for the base/ bottom of the fully-emptied containment system? **[Multiple response]** | |  | | - No material – open base/ bottom | |  |
|  |  |  |  | | - Brick bats or aggregates or sand | |  |
|  |  |  |  | | - Brick with cement | |  |
|  |  |  |  | | - Stone/rubble with cement | |  |
|  |  |  |  | | - Pre-cast RCC Slabs | |  |
|  |  |  |  | | - Reinforced Cement Concrete (RCC) | |  |
|  |  |  |  | | - Fiber Reinforced Plastic or hard plastic | |  |
|  |  |  |  | | - Others, (specify): _______________ | |  |
|  | Is the bottom/ base floor of the containment system fully sealed/ impermeable/ plastered? | |  | | - Yes | |  |
|  |  |  |  | | - No | |  |

Table S11(Cont’d): Observation Survey Checklist

| **Qn. No** | **Question** |  | | **Response** | |
| --- | --- | --- | --- | --- | --- |
|  | Are there partition walls in containment system? | |  | | - Yes |
|  |  |  |  | | - No |
|  |  |  |  | | - Others (specify): ________________ |
|  | How many chambers/ Compartments are there? | |  | | - One |
|  |  |  |  | | - Two |
|  |  |  |  | | - Three |
|  |  |  |  | | - Four |
|  |  |  |  | | - Others (specify): ______________________ |
|  | Was the containment system constructed with an inlet pipe to the containment system? | |  | | - Yes |
|  |  |  |  | | - No |
|  |  |  |  | | - Others (specify): ______________________ |
|  | Was the tank constructed with a plastic sanitary elbow or Tee-pipe inlet pipe? | |  | | - No |
|  |  |  |  | | - Yes |
|  | Was the containment system constructed with an effluent/ outlet pipe? | |  | | - Yes |
|  |  |  |  | | - No |
|  |  |  |  | | - Others (specify) ________________________ |

|  | Where does the effluent pipe from the containment system discharge to? |  | - Soakaways |
| --- | --- | --- | --- |
|  |  |  | - Drain field |
|  |  |  | - Environment -Open ground |
|  |  |  | - Environment – Open drain |
|  |  |  | - Environment - Surface water (Stream, pond or river) |
|  |  |  | - Others (specify) _______________ |
|  | Was the containment system (tank) constructed with a plastic sanitary elbow or Tee-pipe effluent pipe? |  | - No |
|  |  |  | - Yes |
|  | Are there baffle walls in containment system (tank)? |  | - Yes |
|  |  |  | - No |
|  |  |  | - Others (specify): __________________ |
|  | Are the inlet and outlet pipes of the containment system properly configured? |  | - Yes |
|  |  |  | - No |
